# Rice stripe mosaic virus M protein antagonizes G-protein-induced antiviral autophagy in insect vectors

**DOI:** 10.1371/journal.ppat.1013070

**Published:** 2025-04-29

**Authors:** Ruonan Zhang, Tengfei Wang, Yu Cheng, Jiaxin Qiu, Dongsheng Jia, Hongyan Chen, Taiyun Wei, Xiao-Feng Zhang

**Affiliations:** 1 State Key Laboratory of Agriculture and Forestry Biosecurity, Institute of Plant Virology, Fujian Agriculture and Forestry University, Fuzhou, Fujian, China; 2 Spice and Beverage Research Institute, Chinese Academy of Tropical Agricultural Sciences, Wanning, Hainan, China; University of California, Davis Genome Center, UNITED STATES OF AMERICA

## Abstract

In the field, 80% of plant viruses are transmitted by insect vectors. When ingested by a sap-sucking insect such as *Recilia dorsalis*, persistently transmitted viruses such as rice stripe mosaic virus (RSMV) infect the gut epithelium and eventually pass to the salivary glands where they will be transmitted to the next rice (*Oryza sativa*) plant. To efficiently exploit insect vectors for transmission, plant viruses must overcome various immune mechanisms within the vectors, including autophagy. However, understanding how plant viruses overcome insect autophagic defenses remains limited. In this study, we provide evidence that infection with RSMV triggers an autophagic antiviral response in leafhopper cells. In this response, the G protein of RSMV binds to a leafhopper AMP-activated protein kinase (AMPK), leading to enhanced phosphorylation of Beclin-1 (BECN1), thereby inducing autophagy. Knockdown of *AMPK* and genes encoding members of the phosphoinositide 3-kinase (PI3K) complex composed of the autophagy-related protein 14 (ATG14), BECN1, and vacuolar protein sorting 34 (VPS34) facilitated viral infection in leafhoppers. To suppress leafhopper-induced autophagy, RSMV M protein specifically interacts with ATG14, resulting in the disintegration of PI3K complexes. This leads to reduced phosphatidylinositol-3-phosphate content and thus inhibits the G-protein- induced autophagy. Our study sheds light on the mechanism by which this rice virus evades insect autophagy antiviral defenses.

## Author summary

In the field, most plant viruses are transmitted by insects. For example, rice stripe mosaic virus (RSMV) is ingested by the sap-sucking insect *Recilia dorsalis* and eventually infects its salivary glands, enabling transmission to the next rice plant. To successfully use insect vectors for spreading, plant viruses must overcome various immune defenses within the insects, including autophagy. However, the specifics of how plant viruses bypass these defenses are not well understood. Our study reveals that RSMV infection triggers an autophagic antiviral response in the leafhopper, *Recilia dorsalis*. The viral G protein interacts with an insect protein, AMPK, leading to increased autophagy. Conversely, the virus’s M protein suppresses this response by disrupting key autophagy-related complexes. This research provides new insights into the strategies plant viruses use to evade insect immune defenses, enhancing our understanding of virus-vector interactions.

## Introduction

Agricultural pests such as aphids, whiteflies, leafhoppers, planthoppers, and thrips, not only directly feed on crops but also transmit other pathogens, resulting in substantial economic losses to agriculture[1–4]. Among the 65 genera of vector-borne plant viruses, 54% are persistently transmitted by sap-sucking insects [5–9]. As the insect feeds on infected plants, persistently transmitted viruses enter the insect midgut epithelium, establishing an initial infection that subsequently spreads to the hemolymph and ultimately reaches the salivary glands to be horizontally transmitted to healthy plants [10–12]. To infect insect vectors, these plant viruses must overcome multiple tissue and membrane barriers and contend with various insect immune mechanisms, including small interfering RNAs, immune deficiency (IMD), Toll pathway, Janus tyrosine kinase/signal transducer and activator of transcription (JAK-STAT), melanization, and autophagy [13–17]. Growing evidence suggests a pivotal role for autophagy in the infection of insect vectors with vector-borne plant viruses [18–20]. However, our understanding of how these viruses activate and evade autophagy responses in insect vectors remains limited.

Autophagy, a highly conserved cellular process, serves as a critical mechanism for eliminating damaged organelles and intracellular materials in response to diverse stresses [21,22]. The complete autophagy involves three major groups of autophagy-related (ATG) proteins: ATG9 and the ATG1/ATG13 kinase complex, ubiquitin-like complexes (ATG8-PE and ATG5-ATG12-ATG16), and phosphatidylinositol-3-kinase (PI3K) complexes [23,24]. Autophagic flux, the dynamic process of autophagy, is primarily activated by nutrient deprivation and is intricately regulated by AMPK, a central energy sensor. AMPK directly phosphorylates and activates BECN1, thereby enhancing the activity of the VP34-Beclin-1-ATG14 complex [25,26]. This core PI3K complex, localized to the endoplasmic reticulum via ATG14, generates phosphatidylinositol-3-phosphate (PI3P), which is crucial for initiating autophagosome membrane formation [27,28]. ATG4 cleaves a C-terminal arginine residue from nascent ATG8 to form ATG8-I during the initial steps of autophagosome formation. ATG8-I subsequently binds to phosphatidylethanolamine (PE) to form ATG8-II. The subsequent interaction of ATG8 with key autophagy-related proteins facilitates autophagosome assembly and the selective recruitment of cargo [26]. The mature autophagosomes ultimately merge with lysosomes for degradation. However, pathogens often exploit or obstruct this process in order to evade their own autophagic clearance, ensuring their replication and proliferation.

The role of autophagy as a defense mechanism against viral infection in plants has been extensively studied, whereas its role during the infection of insect vectors with vector-borne plant viruses has received limited attention. Unlike its antiviral role in plants, various studies have revealed that several vector-borne plant viruses can also activate autophagic responses in insect vectors; however, they exploit these responses to facilitate viral invasion. We previously demonstrated that non-structural protein P7-1 of southern rice black-streaked dwarf virus (SRBSDV) interacts with the mitochondrial autophagy receptor BCL2 interacting protein 3 (BNIP3), sequestering mitochondria within autophagosomes to prevent apoptosis and promote viral propagation in white-backed planthopper (*Sogatella furcifera*) [19]. SRBSDV P10 protein binds LAMP1 to prevent fusion between the autophagosome and lysosome [29]. The non-structural protein Pns11 of rice gall dwarf virus (RGDV) binds with ATG5 to form incomplete autophagosomes that envelope RGDV virions, facilitating viral spread through the midgut and salivary gland barriers and enhancing viral propagation within *Recilia dorsalis* leafhopper [30,31]. P2 of rice gall dwarf virus induces the formation of autophagosomes, which encapsulate and persistently transport virions within insect vectors. P2 directly recruits the GAPDH-ATG4B complex to induce the formation of initial autophagosomes while preventing ATG14-SNARE proteins from mediating autophagosome–lysosome fusion [20].

Several studies have shown that autophagic responses in insects can suppress plant virus infections. For instance, the infection of whiteflies (*Aleyrodidae*) with tomato yellow leaf curl virus (TYLCV) activates the autophagy pathway, thereby inhibiting viral transmission [32]. Similarly, P10, a major outer capsid protein of Rice black-streaked dwarf virus (RBSDV), induces autophagy in the small brown planthopper (*Laodelphax striatellus*) by promoting GAPDH phosphorylation [33]. Notably, RBSDV binds to PtdIns(3,5)P2 and increases its levels via P10, its primary capsid protein, which inhibits autophagy and facilitates viral propagation [34]. However, the role of autophagy in defending against RSMV and the mechanisms by which RSMV evades autophagic responses in insect vectors remain poorly understood.

RSMV is a cytoplasmic rhabdovirus that poses a significant constraint to rice production. It is persistently transmitted in the field by *R. dorsalis*. The negative single-stranded RNA genome of RSMV encodes seven proteins: nucleoprotein (N), phosphoprotein (P), nonstructural protein P3, viral matrix protein (M), glycoprotein (G), nonstructural protein P6, and the large polymerase protein (L), arranged in the order 3’-N-P-P3-M-G-P6-L-5’ [35]. Among these, G protein interacts with SnRK1/AMPK proteins to induce antiviral autophagy in rice plants [36]. Our previous work demonstrated that RSMV infection also triggers a complete antiviral autophagic response in *R. dorsalis* [37]. However, the mechanisms by which RSMV induces autophagy in insect vectors and how it evades this antiviral response to promote efficient transmission remain unresolved.

In this study, we found that the RSMV G protein induces autophagy in insect cells by interacting with AMPK and promoting BECN1 phosphorylation. To sustain infection in insects, the RSMV M protein competitively binds to ATG14, disrupting the ATG14–BECN1 interaction. This interference affects PI3K polymerization and decreases PI3P levels, thereby inhibiting the autophagy triggered by the G protein. Our findings present a model illustrating the interaction between this vector-borne plant virus and its insect vector, highlighting the critical role of autophagy modulation in their dynamic relationship.

## Results

### Complete autophagy in leafhopper cells induced by RSMV functions as an antiviral defense mechanism

**Fig 1:**
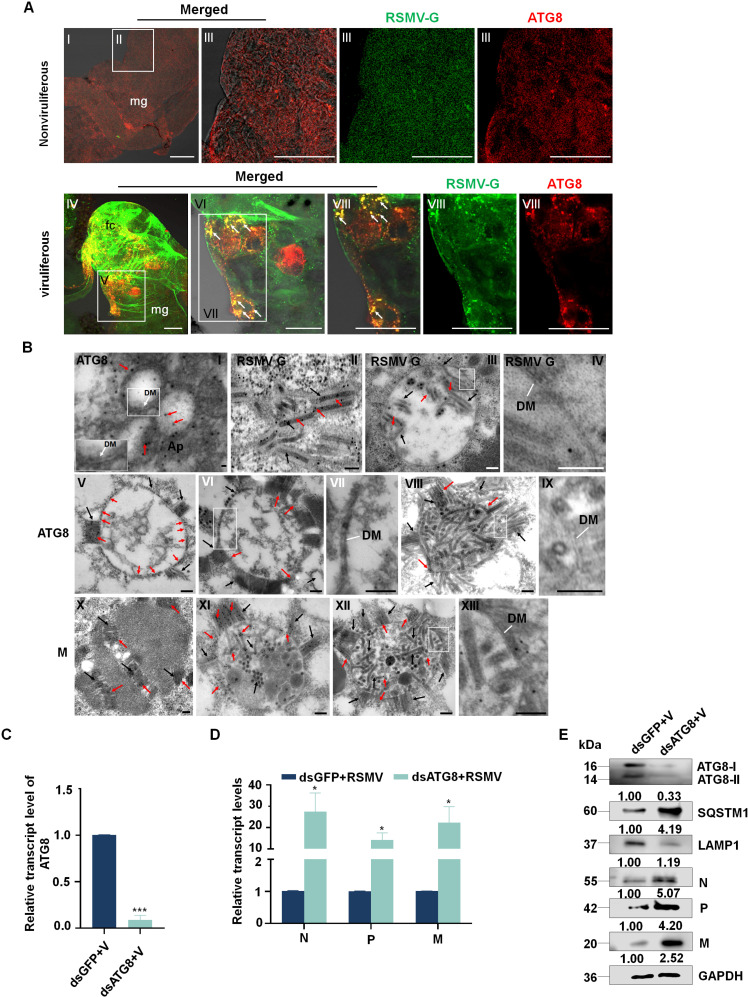
RSMV infection induces complete autophagy in the midgut epithelium of *R. dorsalis.* (A) Immunofluorescence assay showing the colocalization of RSMV-ATG8 in nonviruliferous (panels I-III) or viruliferous (panels IV-VIII) *R. dorsalis* midguts. Intestine tissues of *R. dorsalis* were fixed, immunostained with ATG8-FITC (green) and RSMV-G-rhodamine (red) and observed by immunofluorescence microscopy. Panels III, VI, and VIII are the enlarged images of the boxed areas in panels II, V, and VII, respectively. Arrows indicate the colocalization of RSMV G and ATG8. mg, midgut; fc, filter chamber. Bars: 20 μm. (B). Immunoelectron microscopy showing the localization of ATG8, G, or M on autophagosomes in the cytoplasm of epithelial cells of nonviruliferous or viruliferous *R. dorsalis* midguts, as determined by immunogold labeling. (II-XIII) Intestine tissues of viruliferous *R. dorsalis* were immunolabeled with G-(II-IV), ATG8-(V-IX), and M-(X-XIII) specific IgG primary antibodies, followed by treatment with goat antibodies against rabbit IgG conjugated with 10-nm-diameter gold particles. Panel II displays RSMV virions immunolabeled with a G-specific antibody. Panels III shows RSMV virions localized around autophagosomes, also immunolabeled with the G-specific antibody. Panels V to IX show the process of RSMV virion entry into autophagosomes, as identified by immunolabeling with an ATG8-specific antibody. Panels X to XIII show the viroplasm of RSMV and RSMV virions positioned around autophagosomes, immunolabeled with an M-specific antibody. Panels IV, VII, IX, and XIII are magnified views of the boxed regions in Panels III, VI, VIII and XII, respectively. Red arrows indicate gold particles, black arrows indicate virus, Ap: autophagosome; DM: Double membrane. Bars: 100 nm. (C and D) The silencing efficiency of *ATG8* and viral transcript levels in ds*ATG8*-treated viruliferous individuals, as detected by RT-qPCR at 3 days post-microinjection with ds*ATG8* or ds*GFP*. Means (± SE) from three biological replicates are shown. *P < 0.05, ***P < 0.001. (E) Knockdown of *ATG8* expression promotes RSMV infection and downregulates ATG8 accumulation, as determined by western blot. ATG8, SQSTM1, LAMP1, and RSMV N, P, and M levels in ds*ATG8*- or ds*GFP*-treated *R. dorsalis* were examined by western blot. Relative intensities of bands for ATG8, SQSTM1, LAMP1, and RSMV N, P, and M are shown below. Bands for GAPDH demonstrate the equal loading of proteins. Data are representative of three biological replicates.

Our previous work demonstrated that RSMV-induced autophagy serves as a key antiviral defense mechanism in insect vectors [37]. To further investigate the antiviral role of autophagy in leafhopper cells during RSMV infection, we immunolabeled the midguts of infected *R. dorsalis* with antibodies against RSMV G protein and the autophagic marker ATG8. Confocal microscopy revealed significant accumulation of ATG8 in RSMV-infected cells, forming distinct punctate structures that co-localized with the G protein (Fig 1A).

We used immunoelectron microscopy to directly visualize autophagic vesicles within *R. dorsalis* midgut cells, which were actively engulfing RSMV viral particles. Seven days after the initial contact of *R. dorsalis* with RSMV-infected rice plants, transmission electron microscopy revealed the presence of typical autophagic vesicles (autophagosomes) in the midgut epithelial cells of *R. dorsalis*. These autophagosomes were selectively labeled with gold particles linked to ATG8 antibodies (Fig 1Bi). In addition, using colloidal gold particles conjugated with M protein or G protein for viral labeling, we observed RSMV intact virions displaying a rod-shaped, enveloped morphology and viroplasm (Fig 1Bii and 1Bx). Furthermore, immunoelectron microscopy using ATG8 antibodies, G antibodies or M antibodies revealed the accumulation of virus particles arranged in a rod-like configuration around the outer membranes of autophagosomes (Fig 1Biii-1Bix and 1Bxi-1Bxiii). Some virus particles breached the outer membrane, entering the autophagosomes, where they underwent fragmentation and subsequent degradation (Fig 1Biii, 1Bvi, 1Bviii, 1Bxi and 1Bxii). These results represent the first observation of the degradation of plant arthropod-borne viruses via autophagy in insect vector cells. To further investigate the activation of autophagy-related genes by RSMV infection, the mRNA expression levels of autophagy-related genes *ATG3*, *ATG4*, *ATG5*, *ATG7*, *ATG8, ATG12*, *ATG13*, *SQSTM1*, and *LAMP1* were assessed by RT-qPCR. Meanwhile, the expression levels of ATG8, SQSTM1, and LAMP1 at the protein level were assessed using Western blotting (S1A and S1B Fig). The results indicated that RSMV infection induces complete autophagy in *R. dorsalis*.

To further substantiate the antiviral function of autophagy in *R. dorsalis*, we knocked down the transcription of *ATG8* via microinjection with double-stranded (ds) *ATG8* RNA to suppress autophagy (Fig 1C). We measured the levels of autophagy and expression of RSMV proteins within RSMV-infected *R. dorsalis* following microinjection of ds*ATG8*. Western blot revealed the inhibited conversion from ATG8-I to ATG8-II and decreased accumulation of LAMP1 protein, an important marker of the lysosomal membrane, whereas SQSTM1 protein, a marker of autophagic flux, accumulation increased (Fig 1E). Consequently, RSMV infection was facilitated by ATG8 knockdown (Fig 1D and 1E). Additionally, the effect of viral infection on the survival rate of *R. dorsalis* over a period of 4–12 days was assessed, and it was found that RSMV infection significantly reduced the survival rate of the insects (S1C Fig). To further validate this finding, viruliferous *R. dorsalis* were microinjected with the autophagy activator rapamycin and the autophagy inhibitor 3-MA (3-Methyladenine). The results provided additional evidence that autophagy plays a critical antiviral role in restricting RSMV infection (S1D Fig). These findings underscore the activation of autophagic responses in *R. dorsalis* by RSMV infection, conferring an antiviral function against RSMV invasion.

### RSMV infection promotes PI3P biosynthesis

**Fig 2:**
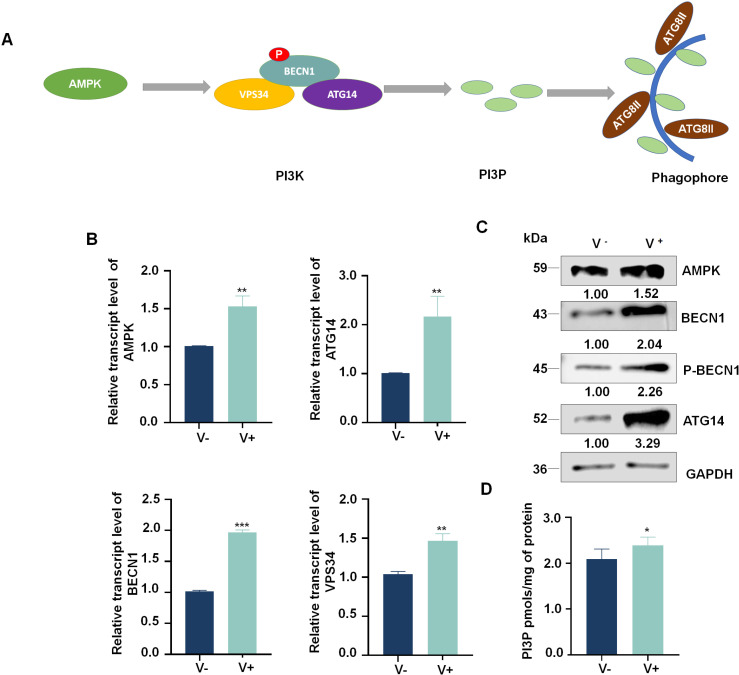
RSMV infection promotes the biosynthesis of PI3P. (A) Model showing that AMPK activates autophagy to generate PI3P by phosphorylating BECN1 in the PI3K complex. PI3P is an important component of the precursor membranes of autophagic vacuoles. (B) Relative transcript levels of *AMPK*, *BECN1*, *ATG14*, and *VPS34* in nonviruliferous or viruliferous insects, as detected by RT-qPCR. (C) Relative levels of AMPK, BECN1, ATG14, and phosphorylated BECN1 in nonviruliferous or viruliferous insects, as detected by western blot. Relative intensities of bands for AMPK, BECN1, p-BECN1, and ATG14 are shown below. GAPDH was used as a control. (D) PI3P content in nonviruliferous or viruliferous *R. dorsalis*, as determined using a PI3P ELISA assay kit. Data are representative of three biological replicates. The raw data are provided in the supplementary material S1 Table. V-: nonviruliferous. V+, viruliferous. *P<0.05, **P<0.01, **, *P*<0.001.

AMPK is an evolutionarily conserved serine/threonine-protein kinase that acts as an energy sensor in cells. Activated AMPK promotes autophagy via the direct phosphorylation of BECN1, a core component of PI3K complexes (ATG14/BECN1/VPS34), to generate PI3P (Fig 2A) [38]. To determine whether RSMV infection increases the expression of *AMPK* as well as genes encoding PI3K complex proteins and the accumulation of their encoded proteins, we performed reverse transcription quantitative PCR (RT-qPCR) and western blot, respectively. Both the RNA and protein levels of AMPK, ATG14, BECN1, and VPS34 were significantly upregulated in viruliferous *R. dorsalis* compared to the control. The phosphorylation of BECN1 and the content of P13P also increased during RSMV infection (Figs 2B-2D and S2A). These results indicate that viral infection promotes the accumulation of AMPK and PI3K complex proteins, thus increasing the accumulation of PI3P to facilitate the autophagy response in insect vectors.

### RSMV G protein induces the antiviral autophagy response in *R. dorsalis*

**Fig 3:**
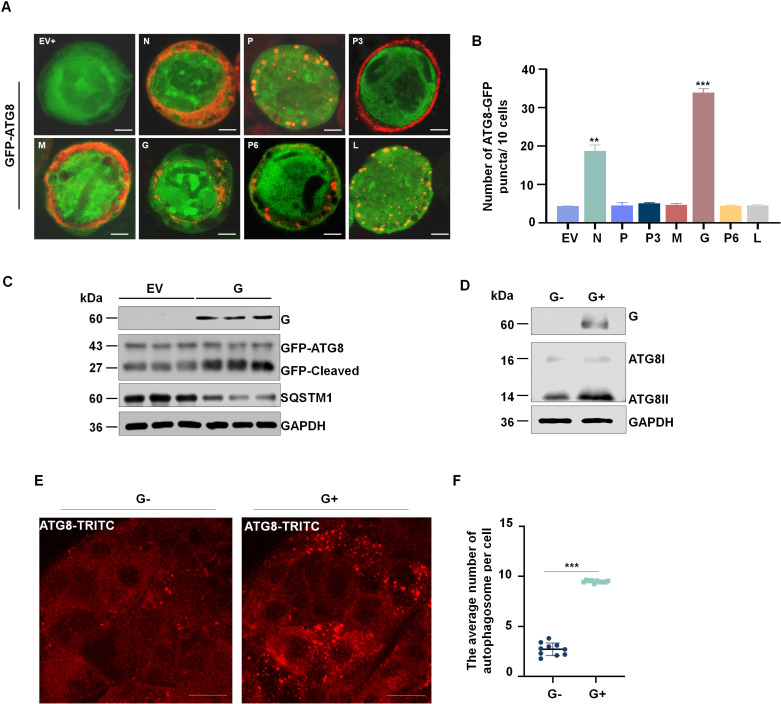
RSMV G protein induces the antiviral autophagy response in the *R. dorsalis* vector. (A) Immunofluorescence assay showing GFP-ATG8 (green) in Sf9 cells co-expressed with an empty vector (EV) as a control, or GFP-ATG8 co-expressed with RSMV proteins (Red) N, P, P3 M, G, P6, or L. Bars, 5 µm. (B) Average number of discrete puncta of GFP-ATG8 in Sf9 cells, as measured in 30 cells. **P < 0.01. (C) ATG8 levels in Sf9 cells expressing G or empty vector, as detected by western blot. GAPDH was used as a control. Data are representative of three biological replicates. (D) ATG8 levels in G protein injected or non-injected insects, as determined by western blot using ATG8-specific IgGs. GAPDH was used as an internal control. Data are representative of three biological replicates. (E) Immunofluorescence assay showing the distribution of ATG8 puncta in midgut epithelial cells of leafhoppers following G protein injection. The intestines from G protein-injected or non-injected *R. dorsalis* individuals were immunostained with ATG8-rhodamine (red), and then examined by immunofluorescence microscopy. Bars, 30 µm. (F) Average number of autophagosomes per cell in midgut epithelial cells from G protein-injected or non-injected insects. Bars represent means ± SE from more than 10 individual cells. ***P<0.001. G−, G protein non-injected. G+, G protein injected.

To investigate how RSMV infection activates autophagy in insect cells, we employed the baculovirus expression system to co-express RSMV-encoded proteins (N, P, P3, M, G, P6, and L) with GFP-ATG8 in *Spodoptera frugiperda* (Sf9) cells. At 3 days post-infection (dpi), confocal microscopy revealed a substantial concentration of autophagic foci formed by GFP-ATG8 in Sf9 cells co-expressing G or N (Fig 3A). However, the average number of discrete GFP-ATG8 puncta was significantly higher when GFP-ATG8 was co-expressed with protein G compared to its co-expression with other RSMV proteins (Fig 3B). When GFP-ATG8, associated with autophagosomes, is delivered to lysosomes for degradation, the resulting free GFP can be detected via Western blot to monitor autophagosome degradation [39,40]. Western blot analysis revealed that co-expression of G with GFP-ATG8 resulted in an increased accumulation of free GFP protein (Fig 3C). These results suggest that protein G induces the activation of complete autophagy in insect cells.

To further confirm the impact of G protein on the induction of autophagy in *R. dorsalis*, we microinjected purified G protein into third-instar nymphs. The microinjection of G protein increased the conversion of ATG8-I to ATG8-II (Fig 3D), The expression level of SQSTM1 protein is reduced (S2B Fig), as revealed by western blot. Thus, G protein alone increased the autophagic flux to induce complete autophagy. Immunofluorescence microscopy revealed the appearance of typical ATG8 puncta in the insect midgut following the microinjection of G protein (Fig 3E). Moreover, the average number of ATG8 puncta per cell was higher after microinjecting G protein (Fig 3F). These findings indicate that RSMV G protein activates autophagic responses in *R. dorsalis*.

### RSMV G triggers autophagy by interacting with AMPK to enhance the phosphorylation of BECN1

**Fig 4:**
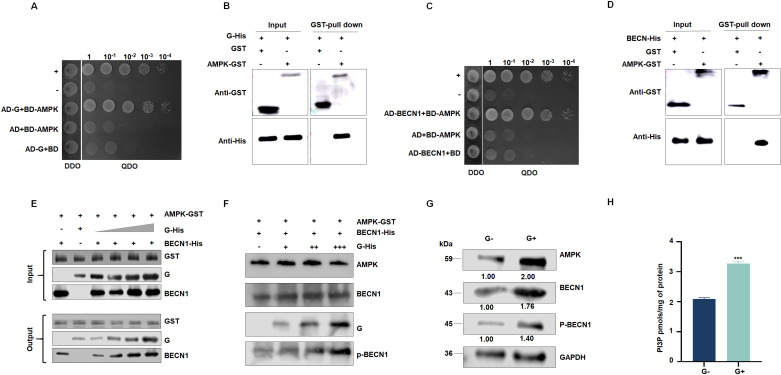
RSMV G interacts with AMPK to enhance the phosphorylation of BECN1. (A and C) Y2H assays showing the interaction of AMPK with G (A) or BECN1 (C). Transformants on SD/-Trp-Leu-Ade-His plates are labeled as follows: +, positive control (pGBKT7-53/pGADT7-T); –, negative control (pGBKT7-Lam/pGADT7-T). DDO, SD -Trp -Leu medium; QDO, SD -Trp -Leu -His -Ade medium. (B and D) Interaction between RSMV G (B) or BECN1 (D) and AMPK of *R. dorsalis*, as detected by GST pull-down assay. (E) The effect of RSMV G on the AMPK–BECN1 interaction in vitro. G protein promotes interactions between AMPK and BECN1, as shown by affinity-isolation assays. GST-AMPK and His-BECN1 were incubated with glutathione-Sepharose beads, followed by the addition of His-G. When the amount of His-G increased, the binding between AMPK and His-BECN1 increased. (F) *In vitro* phosphorylation assay using BECN1-specific antibodies to detect the phosphorylation level of BECN1; AMPK and BECN1 were co-incubated as controls to detect phosphorylation. Increasing amounts of G protein were used to detect the degree of BECN1 phosphorylation. As the G protein content increased, the phosphorylation level of BECN1 increased. (G) Relative levels of AMPK, BECN1, and p-BECN1 in G protein-injected or non-injected insects, as detected by western blot. Relative intensities of bands for AMPK, BECN1, and p-BECN1 are shown below. GAPDH was used as a control. Data are representative of three biological replicates. G−, G protein non-injected. G+, G protein-injected. (H) PI3P content in G protein-injected or non-injected *R. dorsalis*, as determined using a PI3P ELISA assay kit. Data are representative of three biological replicates. ***P<0.001.

To further explore how RSMV G protein induces autophagy, we used G as bait to screen a yeast cDNA library from *R. dorsalis*. In a yeast two-hybrid (Y2H) assay, RSMV G interacted with AMPK, a pivotal regulator of autophagy (Fig 4A). This interaction was further confirmed in glutathione S-transferase (GST) pull-down assay (Fig 4B). Confocal laser microscopy analysis revealed the co-localization of G and AMPK in Sf9 cells (S3A Fig). Co-immunoprecipitation (Co-IP) assays were also performed, further supporting their direct interaction (S3B Fig). These results suggest that RSMV G interacts with AMPK directly.

AMPK, a conserved serine/threonine-protein kinase that functions as a cellular energy sensor, activates autophagy by directly phosphorylating BECN1. Since G protein functions as an autophagy inducer, we reasoned that the interaction of G with AMPK might facilitate the phosphorylation of BECN1. To explore this hypothesis, we first confirmed the AMPK–BECN1 interaction via Y2H and GST pull-down assays (Fig 4C and 4D). Laser confocal microscopy revealed the co-localization of AMPK and BECN1 in Sf9 cells (S3A Fig). This interaction was further validated by co-immunoprecipitation (Co-IP) assays, providing strong evidence of their physical association (S3B Fig). Subsequently, we investigated whether RSMV G influences the interaction between AMPK and BECN1 by performing a dose-dependent affinity-isolation assay. As the concentration of G protein increased, the binding level between AMPK and BECN1 gradually increased (Fig 4E). To support the finding that the binding of G protein to AMPK promotes BECN1 phosphorylation, we conducted an *in vitro* phosphorylation experiment. AMPK efficiently phosphorylated BECN1 *in vitro*, which was enhanced by the addition of G protein (Fig 4F).

Considering that BECN1 is a pivotal component of the PI3K complex, which plays a central role in PI3P biosynthesis, we investigated the potential of G protein to modulate PI3P biosynthesis in *R. dorsalis*. Microinjecting G protein into 3rd instar nymphs caused a significant upregulation of *AMPK*, *BECN1* and a significant increase in the accumulation of the encoded proteins, as revealed by western blot (Fig 4G). Notably, the phosphorylation level of BECN1 (p-BECN1) also increased in *R. dorsalis* following microinjection with G protein (Fig 4G). These, in turn, increased the content of PI3P, as measured using a PI3P ELISA kit (Fig 4H). These findings suggest that G protein promotes the phosphorylation of BECN1 through its interaction with AMPK, thereby enhancing PI3P biosynthesis and activating autophagy. To further investigate the role of the G protein in regulating autophagy and viral replication in *R. dorsalis*, purified G protein was microinjected into viruliferous and nonviruliferous insects. RT-qPCR analysis of autophagy-related genes *ATG3*, *ATG4*, *ATG5*, *ATG7*, *ATG8*, *ATG12, ATG13*, *SQSTM1*, and *LAMP1*, revealed that the G protein significantly activated autophagy in *R. dorsalis*, which suppressed viral replication (Figs 4B and S4A). Western blot analysis further demonstrated increased protein levels of ATG8 and LAMP1, along with decreased levels of SQSTM1 in viruliferous insects injected with the G protein. Notably, the expression of the viral RSMV-P protein was also reduced (S4C Fig). These findings collectively indicate that the G protein induces complete autophagy in *R. dorsalis*, which serves as a critical antiviral defense mechanism.

### Disrupting the PI3K complex decreases the antiviral activity of autophagy

**Fig 5:**
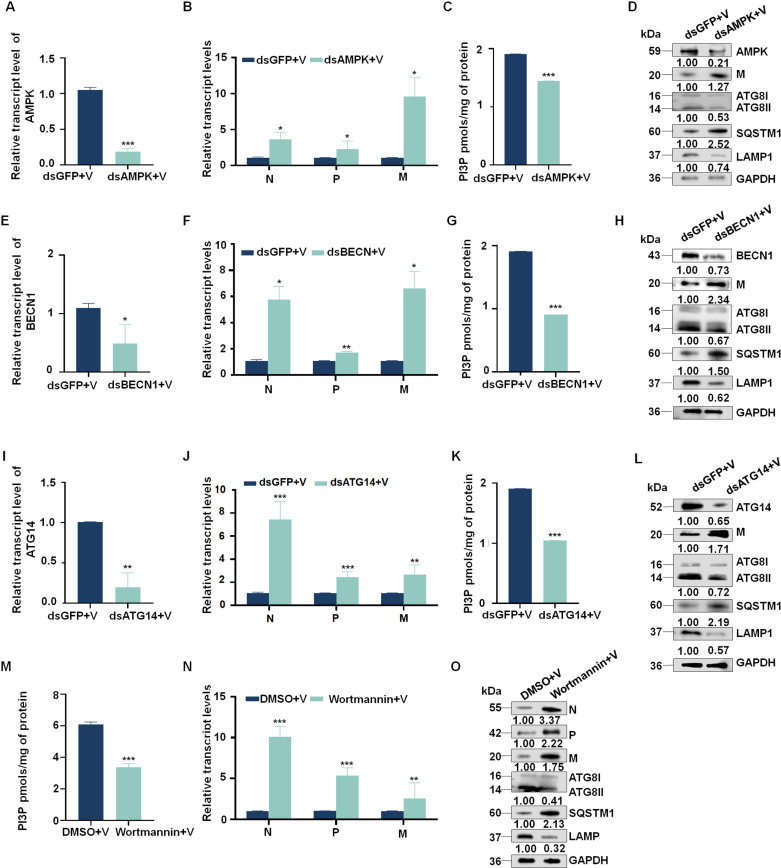
Disrupting the PI3K complex decreases the antiviral activity of autophagy. (A, B, E, F, I, and J) Knockdown of *AMPK*, *BECN1*, and *ATG14* expression promotes the accumulation of *N*, *P*, and *M* mRNA, as determined by RT-qPCR. The relative transcript levels of *AMPK*, *BECN1*, *ATG14*, and *M* in ds*AMPK*-, ds*ATG14*-, ds*BECN1*- or ds*GFP*-treated *R. dorsalis* at 7 days post-first access to diseased plants (padp) are shown. Means (± SE) from 10 *R. dorsalis* are shown. *P<0.05, **P<0.01, ***P<0.001. (C, G, and K) PI3P contents in *R. dorsalis* with knockdown of *AMPK*, *BECN1*, *ATG14*, and ds*GFP*, as determined using a PI3P ELISA assay kit. Data are representative of three biological replicates. ***P<0.001. (D, H, and L) Effect of knockdown of *AMPK*, *BECN1*, and *ATG14* expression on ATG8, SQSTM1, LAMP1, and RSMV M accumulation, as determined by western blot. Relative intensities of bands for AMPK, BECN1, ATG14, SQSTM1, LAMP1, and RSMV M are shown below. Bands for GAPDH demonstrate the loading of equal amounts of protein. Data are expressed as means from three biological replicates. (M) PI3P content in *R. dorsalis* treated with wortmannin or PBS, as determined using a PI3P ELISA assay kit. Data are representative of three biological replicates. (N) Relative transcript levels of RSMV *N*, *P*, and *M* in wortmannin-injected and non-injected insects, as detected by RT-qPCR. Bars represent means ± SE from three independent experiments. **P<0.01, ***P<0.001. (O) Relative levels of ATG8, SQSTM1, LAMP1, RSMV N, P, and M in G protein-injected or non-injected insects, as detected by western blot. Relative intensities of bands for ATG8, SQSTM1, LAMP1, N, P, and M are shown below. GAPDH was used as a control. Data are representative of three biological replicates. V, RSMV.

To investigate the impact of AMPK and the PI3K complex on RSMV infection, we microinjected 3rd instar viruliferous *R. dorsalis* with dsRNA to knock down the expression of *AMPK*, *BECN1*, or *ATG14*. We also microinjected RSMV-infected nymphs with wortmannin (50 nM), a PI3K inhibitor. At 3 days post dsRNA microinjection (dpi), the knockdown of *AMPK*, *BECN1*, and *ATG14* expression significantly decreased PI3P activity as well as the conversion of ATG8-I to ATG8-II and LAMP1 accumulation but increased the accumulation of the viral proteins SQSTM1 and M protein (Fig 5A-5L). Similarly, at 7 days after wortmannin treatment, PI3P activity and the antiviral autophagy response were also inhibited (Fig 5M-5O). To further elucidate the effects of PI3P on autophagy and viral replication, microinjections of PI3P (50 nM) were performed in viruliferous *R. dorsalis*. Western blot analysis revealed significant upregulation of autophagy-related proteins ATG8 and LAMP1, a decrease in SQSTM1 levels, demonstrating that autophagy was significantly activated (S5B Fig). Additionally, both the transcriptional and protein levels of viral components were markedly reduced in PI3P-injected viruliferous *R. dorsalis* (Figs 5B and S5A), suggesting that PI3P enhances autophagy, which in turn acts as an antiviral mechanism. These results suggest that AMPK and PI3K complex proteins play crucial roles in the synthesis of PI3P and are integral to the antiviral autophagy response in insects.

### M protein inhibits the autophagy induced by G protein

**Fig 6:**
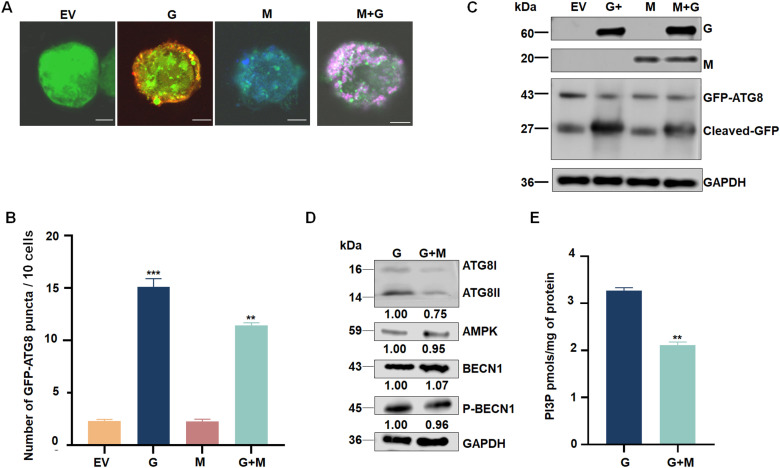
M protein inhibits the autophagy induced by G protein. (A) Immunofluorescence assay showing GFP-ATG8 (green) in Sf9 cells co-expressing empty vector (EV), G (red), or M (blue) with GFP-ATG8 and G plus M with GFP-ATG8. Bars, 5 μm. (B) The average number of discrete puncta of GFP-ATG8 in Sf9 cells. Bars represent means ± SE from more than 10 individual cells. **P<0.01. ***P<0.001. (C) ATG8 levels in Sf9 cells expressing empty vector, G, M, or G plus M, as detected by western blot. GAPDH was used as a control. Data are representative of three biological replicates. (D) Relative levels of ATG8, AMPK, BECN1, and p-BECN1 in insects injected with G protein or G plus M, as detected by western blot. Relative intensities of bands for ATG8, AMPK, BECN1, and p-BECN1 are shown below. GAPDH was used as a control. Data are representative of three biological replicates. (E) PI3P content in *R. dorsalis* injected with G or G plus M, as determined using a PI3P ELISA assay kit. Data are representative of three biological replicates. **P<0.01.

We then investigated how RSMV antagonizes the antiviral autophagy response to facilitate persistent virus infection in insect vectors. Specifically, we investigated whether RSMV protein can suppress the antiviral autophagy response during the viral infection of *R. dorsalis*. We co-expressed GFP-ATG8 with G proteins and other RSMV proteins in Sf9 cells and examined the localization of GFP-ATG8 using confocal microscopy. The average number of GFP-ATG8 puncta was significantly lower when GFP-ATG8 was co-expressed with M or with G and M, compared to when GFP-ATG8 was co-expressed with G alone (Fig 6A and 6B). No significant inhibition of G protein-induced autophagy was observed with the other RSMV proteins (S6 Fig). Western blot analysis revealed that the accumulation of cleaved GFP was lower when GFP-ATG8 was co-expressed with both G and M, compared to when it was co-expressed with G alone (Fig 6C). These findings suggest that M protein effectively suppressed G-induced autophagy in Sf9 cells.

**Fig 7:**
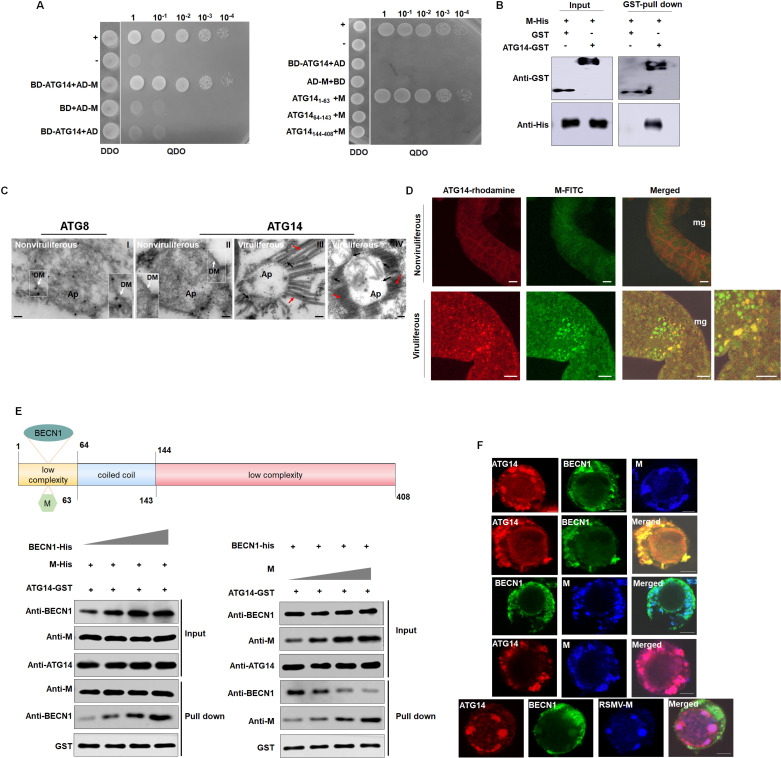
M disrupts the interaction between ATG14 and BECN1. (A) Y2H assays showing the ATG14-N terminal interact with M. Transformants on SD/-Trp-Leu-Ade-His plates are labeled as follows: +, positive control (pGBKT7-53/pGADT7-T); –, negative control (pGBKT7-Lam/pGADT7-T); DDO, SD -Trp -Leu medium; QDO, SD -Trp -Leu -His -Ade medium. (B) Interaction between RSMV M and ATG14 of *R. dorsalis* detected by GST pull-down assay. (C) Immunoelectron microscopy showing the localization of ATG8 and ATG14 on autophagosomes in the cytoplasm of epithelial cells of nonviruliferous or viruliferous *R. dorsalis* midguts, as determined by immunogold labeling. Intestine tissues of nonviruliferous or viruliferous *R. dorsalis* were immunolabeled with ATG8- or ATG14-specific IgG primary antibodies. Panel I show the cytoplasm of epithelial cells of nonviruliferous *R. dorsalis* midguts were labeled with ATG8-specific antibodies. Panel II-IV show the intestine tissues were then treated with ATG14-specific antibodies. Red arrows indicate gold particles, black arrows indicate virus, Ap: autophagosome; DM: Double membrane. Bars: 100 nm (D) Immunofluorescence microscopy of the intestines of co-infected insect vectors immunostained with RSMV ATG14-rhodamine (red) and RSMV M-FITC (green). Mg, midgut. Bars, 50 μm. (E) Schematic diagram showing that the N-terminus of ATG14 interacts with both M and BECN1. Competitive interactions occurred among M, BECN1, and ATG14, as revealed by affinity-isolation assays. GST-ATG14 and BECN1-His were incubated with glutathione-Sepharose beads, followed by the addition of M-His. The amounts of BECN1-His decreased with increasing binding between ATG14 and M. When the amount of BECN1-His increased, the binding between ATG14 and M was not affected. (F) ATG14, BECN1, and M were expressed alone or co-expressed. Sf9 cells were fixed at 48 hpi and immunolabeled with ATG14- rhodamine (red), BECN1-FITC (green), or M-Alexa Fluor 647 (blue). The images showing single expression or co-expression were merged under a background of transmitted light. Bars: 10 μm.

Furthermore, we microinjected purified M protein in combination with G protein into *R. dorsalis* larvae. Western blot assays showed that the co-microinjection of M with G effectively suppressed G protein-induced autophagy, as evidenced as decreasing ATG8-I to ATG8-II conversion (Fig 6D). Interestingly, co-microinjection of M with G did not significantly impair the protein expression of the G protein-activated AMPK and PI3K complex, yet it significantly reduced the PI3P content (Fig 6D and 6E). These findings suggest that the inhibition of autophagy by M protein may occur upstream in the autophagy pathway, potentially involving PI3K formation and PI3P biosynthesis. To further investigate the effects of the M protein on autophagy and viral infection, purified M protein was microinjected into viruliferous insects. RT-qPCR was used to examine the mRNA transcription levels of the PI3K complex genes (*VPS34*, *ATG14, BECN1*), *AMPK*, *ATG8*, *LAMP1*, *SQSTM1*, and RSMV-*N*, RSMV-*P*, and RSMV-*M*. The results indicated that the M protein did not affect the transcriptional or protein levels of the PI3K complex genes (VPS34, ATG14, BECN1) and AMPK (Figs 7D and S7A). However, it significantly suppressed the transcription and protein expression levels of autophagy-related genes ATG8, LAMP1, while promoting the accumulation of RSMV-N, RSMV-P, and RSMV-M (S7B and S7D Fig). Notably, microinjection of the M protein does not affect the transcriptional levels of SQSTM1; however, it significantly increases the accumulation of SQSTM1 protein (Figs 7D and S7B). These findings demonstrate that the M protein inhibits autophagy and facilitates viral replication. Additionally, when M protein was microinjected into nonviruliferous insects, Western blot analysis and PI3P quantification assays revealed that the M protein reduced PI3P levels as well as the expression of autophagy-related genes ATG8 and LAMP1 (S7E and S7F Fig). However, it did not affect the expression of AMPK or PI3K complex genes such as ATG14 and BECN1 (S7E Fig). These results indicate that the M protein does not alter the expression of the PI3K complex genes but suppresses the production of PI3P, thereby inhibiting autophagy. Meanwhile, to identify the time points at which G and M proteins influence autophagy, RT-qPCR was performed to monitor the transcriptional levels of ATG8, LAMP1, SQSTM1, RSMV-M, and RSMV-G in viruliferous and non-viruliferous insects between 4- and 12-days post-acquisition (padp) (S8 Fig). The results revealed an initial increase in autophagy levels with viral infection, followed by a decline between 6 and 8 padp. These findings suggest that G protein induces autophagy at 4 padp, while the M protein inhibits autophagy during the 6–8 padp.

### M disrupts the interaction between ATG14 and BECN1

To elucidate the molecular mechanism behind the role of M protein in inhibiting autophagy, we employed M protein as a bait to screen a yeast cDNA library from *R. dorsalis* via Y2H screening, finding that the N-terminal region of *R. dorsalis* ATG14 bound to M protein. Subsequently, we cloned full-length *ATG14* based on a bioinformatics analysis of *R. dorsalis* RNA-seq data. We confirmed the interaction between M and ATG14 through Y2H and GST pull-down assays (Fig 7A and 7B). ATG14 is an essential component of the PI3K complex involved in the initiation of autophagy [39]. Therefore, we reasoned that the binding of M protein to ATG14 might interfere with the interaction between ATG14 and other PI3K complex proteins, such as BECN1 or VPS34.

To confirm the interactions between ATG14, BECN1, and VPS34, we performed Y2H assays. ATG14 was found to interact with BECN1, with the N-terminal region (1–63 aa) of ATG14 identified as the key region mediating this interaction. Additionally, BECN1 interacted with VPS34, whereas ATG14 did not show any interaction with VPS34. These findings were further validated by GST pull-down assays (S9 Fig). Using immunoelectron microscopy, we observed ATG14 localization on viral particles at the outer membrane of the autophagosomes. Laser-scanning confocal microscopy confirmed the colocalization of ATG14 with M antibody-labeled viral particles (Fig 7C and 7D). These results indicate that M protein interacts with ATG14 within the *R. dorsalis*.

Both M and BECN1 interacted with the N-terminus of ATG14, suggesting that M protein and BECN1 might compete for binding to ATG14. To test this hypothesis, we conducted *in vitro* protein competition experiments. The binding capacity of ATG14 to BECN1 decreased with increasing levels of M protein. However, when the levels of BECN1 protein increased, there was no significant change in the binding of ATG14 to M protein (Fig 7E). Therefore, RSMV M competitively interfered with the interaction of ATG14 with BECN1.

Finally, we investigated the interaction of M, ATG14, or BECN1 in Sf9 cells. When ATG14 and BECN1 were co-expressed in Sf9 cells, they colocalized to the cytoplasm. However, when M, BECN1, and ATG14 were simultaneously expressed in Sf9 cells, ATG14 formed a complex with M within the cytoplasm, whereas the colocalization of ATG14 and BECN1 was not observed (Fig 7F). Together, these results suggest that M competes for binding with ATG14, obstructing its interaction with BECN1, thus disrupting the formation of the PI3K complex and decreasing the biosynthesis of PI3P, thereby inhibiting autophagy.

## Discussion

Autophagy serves as a crucial defense mechanism against intracellular pathogens in metazoan and plant systems [40–42]. Pathogens employ various strategies to evade or suppress host cell autophagy. Recent studies have highlighted the capability of vector-borne plant viruses to induce autophagic responses in insect vector cells, thereby contributing to viral infection [15,20,41]. For example, RGDV induces pro-viral autophagy and exploits autophagosomes for viral accommodation and dissemination in leafhopper [30]. RGDV P2 mediates the binding of GAPDH to ATG14 and inhibits the interaction of ATG14 with SNAP29, thus preventing ATG14-SNARE proteins from mediating autophagosome-lysosome fusion and facilitating persistent viral propagation in insect vectors [20]. Southern rice black-streaked dwarf virus (SRBSDV) can trigger mitophagy in its planthopper vector to prevent mitochondria-dependent apoptosis and promote persistent viral propagation [19]. However, the specific mechanisms by which viruses resist or modulate autophagy and immune responses remain largely unexplored.

**Fig 8:**
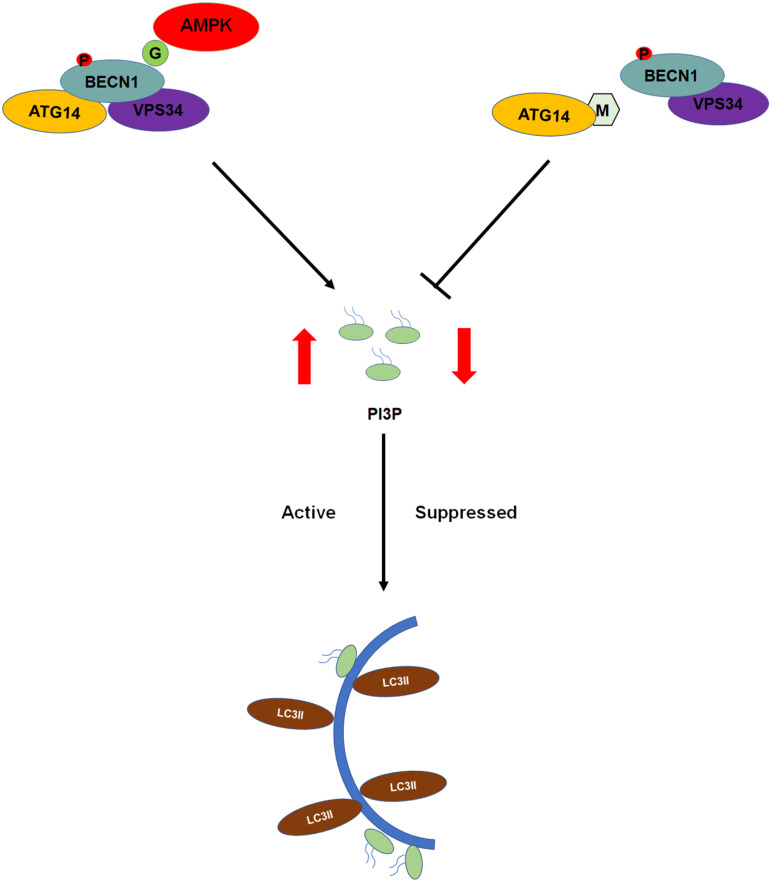
Proposed model of the induction and suppression of autophagy in insect vectors during RSMV infection. According to the model, the interaction between RSMV G protein and AMPK facilitates the phosphorylation of BECN1, triggering antiviral autophagy in *R. dorsalis*. To sustain viral infection, RSMV M protein competitively binds with ATG14, disrupting the PI3K complex and inhibiting autophagy, thereby promoting viral replication.

In this study, we demonstrated that RSMV G protein can activate an antiviral autophagic response in insect cells by recruiting AMPK, a known activator of autophagy. Intriguingly, under field conditions, RSMV is efficiently transmitted through persistent proliferation in insect vectors, suggesting that the virus has evolved mechanisms to evade autophagy. Our analysis identified the RSMV matrix protein M as an autophagy suppressor. M protein interacts with ATG14, disrupting the formation of the PI3K complex and inhibiting the process of autophagy (Fig 8). These findings elucidate how a vector-borne plant virus activates autophagy in vector cells while suppressing cellular autophagy, offering a new model for understanding the persistent transmission of RSMV by *R. dorsalis*.

AMPK serves as a critical regulator of cellular energy homeostasis, playing a pivotal role in autophagy. Upon activation, AMPK directly stimulates autophagy by phosphorylating key components within the autophagy initiation complex, including unc-51-like (ULK1) and Beclin-1. Several viruses, such as hepatitis C, influenza A, and dengue virus, have been shown to induce autophagy through AMPK activation [43–45]. Notably, RBSDV P10 interacts with AMPK and GAPDH, resulting in their phosphorylation. The phosphorylated GAPDHs then translocate into the nucleus, thereby activating the autophagy in *L. striatellus* [19]. Importantly, our findings reveal that RSMV G protein can also activate autophagy in insect cells. Consistent with its mechanism in inducing autophagy in rice, G protein is recognized by AMPK, promoting BECN1 phosphorylation and facilitating the occurrence of autophagy. Silencing of *AMPK* and *BECN1* inhibited autophagy, thereby augmenting RSMV infection in insect vectors. These findings underscore the crucial role of AMPK, whether in plant or insect cells, in sensing viral infection. Through binding to virus-encoded proteins, AMPK plays a vital role in initiating the phosphorylation of downstream key proteins, thereby coordinating the antiviral autophagy process.

Autophagy functions as a double-edged sword, as it can both suppress pathogen invasion and, if uncontrolled, inflict severe damage to host cells. To successfully infect hosts, many plant virus proteins inhibit autophagy in host plant cells by binding to autophagy-related proteins [41,46]. Our findings provide the first evidence that RSMV M protein can suppress autophagy induced by G protein in *R. dorsalis* cells. RSMV M protein binds to ATG14 and impedes its interaction with BECN1. ATG14 and BECN1 are crucial components of the PI3K complex, which plays a central role in the early stages of autophagy by generating PI3P and coordinating the recruitment of essential autophagy-related proteins. Our results also demonstrate that M protein can reduce PI3P biosynthesis. These findings suggest that M protein disrupts the formation of the PI3K complex, thereby affecting PI3P dynamics and inhibiting the autophagy process.

In this study, we validated the ability of *R. dorsalis* AMPK to recognize RSMV G protein and instigate cellular autophagy as a means of suppressing viral infection. To extend the viability of insects as vectors for viral transmission, the RSMV-encoded M protein disrupts the interaction between ATG14 and BECN1, thereby impairing the PI3K complex and hindering autophagy initiation. While our study has provided valuable insights, several issues warrant further investigation. For example, although we observed the encapsulation of RSMV viral particles by autophagosomes, the mechanisms governing the entry of RSMV viral particles into autophagosomes remain unclear. Additionally, unraveling the coordinated and dynamic regulation of autophagic responses by G and M proteins during RSMV infection in *R. dorsalis* cells require further exploration. A comprehensive understanding of how these proteins ensure the enduring symbiosis between RSMV and insect vectors is crucial.

In the insect organism like *D. melanogaster*, autophagy stands out as a highly conserved process driven by key protein complexes. It begins with the Atg1/ULK complex, which initiates autophagosome formation during the induction phase. The PI3K complex then mediates vesicle nucleation by recruiting essential proteins to the phagophore assembly site (PAS). Subsequent phagophore expansion is facilitated by the Atg9/Atg2/Atg18 cycling system, while two ubiquitin-like conjugation systems (Atg5-Atg12 and Atg8) promote vesicle expansion, culminating in autophagosome maturation. Our research on *R. dorsalis* reveals that its autophagy pathway is largely similar to that of *D. melanogaster* [47]. However, a few subtle but notable differences have been identified. Autophagy is regulated by the mono-allelic expression of AMPK subunits (AMPK-α, -β, and -γ), which together form a heterotrimeric energy sensor responsible for maintaining cellular energy homeostasis in *D. melanogaster* [48]. However, only the AMPK-α gene is present in *R. dorsalis*, with no evidence of the other subunit alleles, indicating a divergence in energy-sensing mechanisms between the two species. Unlike in *D. melanogaster*, where VPS34 was found to interact with ATG14 [49], such an interaction was not observed between ATG14 and VPS34 in *R. dorsalis* (S9D Fig). These findings emphasize the critical role of BECN1 as a bridging protein that connects ATG14 and VPS34 during PI3K complex assembly, a process essential for PAS formation and autophagosome biogenesis in *R. dorsalis*. Despite these subtle differences, the overall autophagy pathways in *R. dorsalis* and *D. melanogaster* are highly similar, highlighting species-specific adaptations that warrant further investigation. In summary, our findings provide important details about how vector-borne plant viruses modulate autophagy in insect vector cells, offering a model for investigating the transmission mechanisms of vector-borne plant viruses through insect vectors.

## Materials and Methods

### Plasmid Construction

All primers used for cloning and experiments are listed in S2 Table. All plasmids constructed in this study are listed in S3 Table, Enzymes from Takara (Cat No. R010A) and enzymes from Genstar (Cat No. A002-10) are used for PCR amplification and verification. Enzymes from Takara (Cat No. 1611, 1605, 1615) and enzymes from Genstar (Cat No. T197-100) are used for nucleic acid digestion and ligation.

### Viruses, insects, cells, and antibodies

Nonviruliferous individuals of *R. dorsalis* were collected in Fujian Province in southern China and propagated and infected with the virus in the laboratory. Rice infected by RSMV was collected in Yunfu, Guangdong Province, China and was transmitted by *R. dorsalis* in the greenhouse. Sf9 cells were cultured and maintained in Sf900 III growth medium (Gibco, 12658019). Specific antibodies against RSMV N, M, and P, ATG8, and SQSTM1 of *R. dorsalis* were prepared as described previously [8,31]. Rabbit polyclonal antibodies against AMPK, BECN1, and ATG14 were prepared by Genscript Biotech Corporation (Nanjing, China), as approved by the Science Technology Department of Jiangsu Province of China. Rabbit polyclonal antibodies against LAMP1 and p-BECN1 were purchased from ABclonal Technology Corporation (Wuhan, China), as approved by the Science Technology Department of Hubei Province of China. Mouse monoclonal antibodies against 6×His tag, Strep, and GST were purchased from TransGen Biotech (HT501 and HT601). The antibodies were conjugated directly to fluorescein isothiocyanate (FITC) (Thermo Fisher Scientific, 46424), rhodamine (Thermo Fisher Scientific, 46406), or Alexa Fluor 647 carboxylic acid (Thermo Fisher Scientific, A33084) according to the manufacturer’s instructions.

### Transmission electron microscopy

The midgut dissected from a viruliferous or nonviruliferous *R. dorsalis* individual was fixed, dehydrated, embedded, and sliced as previously described [50,51]. For immunoelectron microscopy, ultrathin sections were labeled with M-, ATG14-, or ATG8-specific IgG as a primary antibody and treated with goat anti-rabbit IgG conjugated to 10 nm- (Sigma-Aldrich, G7402) as a secondary antibody. The ultrathin sections were observed under a transmission electron microscope (H-7650; Hitachi, Tokyo, Japan).

### Immunofluorescence microscopy

To determine the localization of autophagy proteins and RSMV in the midgut of *R. dorsalis*, the midgut was dissected in PBS, fixed in 4% (v/v) paraformaldehyde (Coolaber Science & Technology, PM5090) for 2 h, and infiltrated in 0.2% Triton X-100 (Vetec, V900502) for 1 h. The midgut tissue was washed with PBS, immunolabeled with ATG8- or M-specific IgG conjugated to FITC or rhodamine and processed for immunostaining. Immunostained samples were analyzed by immunofluorescence microscopy and imaged using a Leica TCS SP5 inverted confocal microscope [52].

### RT-qPCR and immunoblot assays

For quantitative analysis of gene expression, total RNA was extracted from 10 *R. dorsalis*. RT-qPCR assays were performed using 2 × RealStar Fast SYBR qPCR Mix (High ROX) (GenStar, A303). Gene expression levels were calculated by the 2^-ΔΔCT^ method using *elongation factors 1 alpha* (EEF1A/EF-1α) transcript from *R. dorsalis* as an internal reference.

Total protein was extracted from an average of 10 *R. dorsalis* for relative quantification of protein expression. AMPK-, BECN1-, ATG14, ATG8-, SQSTM1-, LAMP1-, and GAPDH-specific IgG served as primary antibodies for western blot, whereas goat anti-rabbit IgG-peroxidase served as the secondary antibody (Sangon Biotech, D110058). Moreover, GST-, 6×His tag-, or GFP-specific IgG served as the primary antibodies, and goat anti-rabbit or anti-mouse IgG-peroxidase served as the secondary antibody (Sangon Biotech, D110103). Protein bands were displayed using Enhanced Chemiluminescence (ECL) Immunoblotting reagent (Thermo Fisher Scientific, 32209). The band intensities of proteins analyzed by immunoblotting were quantified with ImageJ software.

### Yeast two-hybrid assay

Protein–protein interactions were verified by Y2H. The open reading frames (ORFs) of *AMPK* and *ATG14* were separately cloned into pGBKT7 (Clontech, 630489) as bait plasmids. The ORFs of *G*, *M*, and *VPS34* were separately cloned into pGADT7 (Clontech, K1612-1) as prey plasmids. The bait and prey plasmids were co-transformed into yeast strain AH109, and β-galactosidase activity was detected on SD/Leu-Trp-His-Ade-/X-a-gal culture medium (Clontech, 630412) as described previously [53]. Both the pGBKT7–53 and pGADT7-T plasmids were co-transformed into AH109 cells as the positive control, and both pGBKT7-Lam and pGADT7-T were co-transformed into AH109 cells as the negative control.

### GST pull-down assay

To examine interactions of AMPK and ATG14 with the ORFs of *G*, *M*, and *BECN1*, the ORFs of *AMPK* and *ATG14* were cloned into pGEX-4T-3 (constructed in our laboratory) to generate plasmids expressing GST fusion protein as baits for fusion with GST tag as previously described [51]. The ORFs of RSMV *G* and *M* were cloned into pET28b for fusion with His tag. The plasmids expressing His fusion proteins (His-M and -G) were used as preys. The recombinant proteins fused with a GST and His tag were expressed separately in *Escherichia coli* strain BL21 or Rosetta. The lysates were incubated with glutathione-Sepharose beads (GE Healthcare, 17-0756-01) and subsequently incubated with the recombinant proteins fused with His tag. The eluants were analyzed by western blot using GST-tag and His-tag antibodies.

### Baculovirus expression of proteins

Baculovirus expression of GFP-ATG8, ATG14-Strep, BECN1-His, or M-His was performed according to the manufacturer’s instructions (Thermo Fisher Scientific, 10359016). The ORFs of the above genes were retroprimed to add the corresponding nucleic acid sequence of tag protein, the tagged nucleic acid sequences were fused to the amplified ORFs, and the ORFs with the tagged nucleic acid sequences were cloned in the pFAST vector, generating the complete shuttle plasmid. Recombinant bacmids were generated by transforming *E. coli* DH10Bac with the recombinant baculovirus. Recombinant baculovirus plasmids were transfected into Sf9 cells using Cellfectin II reagent (Gibco, 10362100). Infection solution P1 was collected four days post-infection after centrifugation at room temperature at 1000 rpm for 5 minutes and used to re-infect Sf9 cells. After 4 days of incubation, the cells were centrifuged and the supernatant collected. Sf9 cells attached to a cover glass were infected with the supernatant, and samples were prepared to observe immunofluorescence via confocal microscopy at two days after infection.

### PI3P content measure

The total amount of PI3P was measured using a quantitative and competitive ELISA assay, following the manufacturer’s instructions (PI3P ELISA Kit, Product No. ml037511, mlbio, Shanghai, China). Tissue homogenate was extracted from the bodies of an average of 30 *R. dorsalis* and incubated with a PI3P-specific detector protein. The mixture was then transferred to a PI3P-precoated microplate for competitive binding. A peroxidase-conjugated antibody specific to the PI3P detector protein was added, and colorimetric detection was performed to quantify PI3P binding on the plate. Cellular PI3P levels were determined using a standard curve generated through non-linear fitting of PI3P standards. Standard curves for PI3P were established based on OD values and the concentrations of the standards provided in the kit. The standard curve equation was y = 0.3673x – 0.089 (where x represents the concentration gradient of the standards, ranging from 0 to 5 pmols/ml, and y represents the OD value measured at a wavelength of 450 nm, with R² = 0.9917). The PI3P content was calculated as the amount of PI3P in pmol per milligram of insect tissue, based on the measured OD value and the corresponding equation.

### Phosphorylation assay of BECN1 in vitro

The *E. coli* BL21 expression system was used for prokaryotic expression of AMPK-GST and BECN1-His. The proteins were purified using GST and His purification columns. For the BECN1 phosphorylation assay, the relevant fusion proteins were added to reaction buffer (100 mM Tris-HCl pH 7.0, 2 mM DTT, 20 μM ATP, 0.4 mM CaCl_2_, and 20 μM of ATP) and incubated in a water bath at 37°C for 1 h. Western blot was used to detect the phosphorylation of BECN1 using p-BECN1 (ABclonal, AP1287) as the antibody.

### Silencing of *AMPK, ATG14, BECN1, VPS34* or *ATG8* in viruliferous *R. dorsalis*

The T7 RNA polymerase promoter sequence 5’-ATTCTCTAGAAGCTTAATACGACTCACTATAGGG-3’ was combined with specific primers to amplify a region of approximately 1000–1200 bp of the *AMPK*, *BECN1*, *ATG14*, or *VPS34* gene and 500 bp of *GFP* or *ATG8*. The T7 RiboMAX Express RNAi System (Promega, P1700) was used to synthesize dsRNAs targeting *ATG8* (ds*ATG8*), *GFP* (ds*GFP*), *AMPK* (ds*AMPK*), *ATG14* (ds*ATG14*), *BECN1* (ds*BECN1*), or *VPS34* (ds*VPS34*). Nonviruliferous second-instar *R. dorsalis* were infected by RSMV with ds*ATG8*, ds*GFP*, ds*AMPK*, ds*BECN1*, or ds*VPS34* (approximately 200 ng/insect) at the intersegment region of the thorax using a Nanoject II Auto-Nanoliter Injector (Spring). The injected *R. dorsalis* were transferred to healthy rice seedlings.

After 5 days of RNAi treatment, 10 *R. dorsalis* per treatment were collected to comprise one group. At least five groups were examined by RT-qPCR to detect the relative expression levels of the corresponding genes. *EF-1α* was used as the reference gene, and ds*GFP* was used as the control. At the same time, autophagy-related protein levels were examined in the treated *R. dorsalis*: approximately 20 *R. dorsalis* were collected to examine protein levels by western blot.

### Co-IP assay

The ORFs of AMPK-Flag, GFP-Flag, GST-BECN1, GST-G and GST were cloned into the pFAST vector. Recombinant bacmids were generated by transforming *E. coli* DH10Bac with the recombinant baculovirus plasmids. Recombinant baculovirus plasmids were transfected into Sf9 cells were extracted in extraction buffer (10% [v/v] glycerol 25 mM Tris, pH 7.5, 1 mM EDTA, 150 mM NaCl, 1% Tween, and protease inhibitor cocktail). The Sf9 cells were subjected to three cycles of freezing and thawing in liquid nitrogen, with each cycle lasting 2 minutes, and the supernatant was immunoprecipitated with Glutathione Sepharose 4B beads (Amersham, USA) for 3 h at 4 °C The precipitations were washed three times with IP buffer (25 mM Tris-HCl pH 7.5. 150 mM NaCl, 1 mM EDTA,10% [v/vl glycerol, 1% Tween-20, and 10 mM PMSF) at 4°C and analyzed by western blot using with anti-Flag or anti-GFP antibody.

### Quantification and statistical analysis

All quantitative data presented in the text and figures were analyzed with GraphPad Prism 8.0 (GraphPad Software, San Diego, CA, USA). Comparisons between mean values in two groups were conducted using an independent Student’s *t*-test.

## Supporting information

S1 Fig RSMV induces complete autophagy to mediate antiviral responses in *R. dorsalis.* (**A**)Relative transcript levels for *ATG3*, *ATG4*, *ATG5*, *ATG7, ATG8*, *ATG12*, *ATG13, SQSTM1* and *LAMP1* in nonviruliferous and viruliferous insects, as measured by RT-qPCR assay. (**B**) The expressions of ATG8, SQSTM1 and LAMP1 in nonviruliferous or viruliferous insects as detected by western blot assay. GAPDH was detected as a control. Data are representative of three biological replicates. (**C**) The survival rates of viruliferous insects were evaluated after microinjection with ds*GFP* or ds*ATG8*, with 100 insects per group. Means (± SD) from three biological replicates are shown. (**D**) The relative transcript levels of RSMV-*N*, -*P*, and -*M* were determined by RT-qPCR following treatments with rapamycin or 3-MA for 5 days. Means (± SE) from three biological replicates are shown. RP, rapamycin; 3-MA, 3-Methyladenine; *P < 0.05, **P < 0.01, ***P < 0.001.
(TIF)

S2 Fig Induction of autophagy by RSMV or purified G protein in *R. dorsalis.* (A) RSMV infection increases the expression of VPS34 protein in *R. dorsalis*. Relative intensities of bands for of VPS34 are shown below. GAPDH was used as a control. Data are representative of three biological replicates. V-, nonviruliferous, V+, viruliferous. (B) The expression of SQSTM1 in *R. dorsalis* with the microinjection of G. GAPDH was used as a control. Data are representative of three biological replicates. G+, G protein injected, G-, G protein non-injected.
(TIF)

S3 Fig AMPK interacts with BECN1 and G in Sf9 cells (A) AMPK-MYC, BECN1-His, and G-His were expressed alone or co-expressed. Sf9 cells were fixed at 48 hpi and immunolabeled with AMPK- rhodamine (red), BECN1-FITC (green), or G-FITC (green). The images showing single expression or co-expression were merged under abackground of transmitted light. Bars: 10 um. **(B)** Co-IP assays examining the interactions of AMPK/BECN1 and AMPK/G in vivo. At 3 days post-infiltration, total proteins extracted Sf9 cells expressing indicated proteins were precipitated with anti-GST beads and analyzed by western blotting with anti-GST and anti-Flag antibodies.
(TIF)

S4 Fig RSMV G protein induces complete autophagy to mediate antiviral responses in *R. dorsalis.* (**A, B**)Relative transcript levels for *ATG3*, *ATG4*, *ATG5*, *ATG7*, *ATG8*, *ATG12*, *ATG13*, *SQSTM1*, *LAMP1* (A), RSMV-*N*, –*P* and -*M* (B) in viruliferous insects injected with G or without G, as measured by RT-qPCR assay. Means (± SE) from three biological replicates are shown. (**C**) Relative intensities of bands for ATG8, SQSTM1, LAMP1, and RSMV-P are shown below. GAPDH was used as a control. Data are representative of three biological replicates. G, G protein injected; **P < 0.01, ***P < 0.001.
(TIF)

S5 Fig Effects of microinjected PI3P on autophagy and RSMV replication in *R. dorsalis.* (**A**)The relative transcript levels of RSMV-*N*, -*P*, and -*M* were determined by RT-qPCR following microinjected with PI3P or without PI3P for 7 days. Means (± SE) from three biological replicates are shown. Data are presented as mean (± SE). *P<0.05, ***P<0.001. (**B**) Relative intensities of bands for of RSMV-N, -P, -MATG8, SQSTM1 and LAMP1 are shown below. GAPDH was used as a control. Data are representative of three biological replicates.
(TIF)

S6 Fig M protein inhibits the autophagy induced by G protein. (**A**) Immunofluorescence assay showing GFP-ATG8 (green) in sf9 cells co-expressed with G as a control, or GFP-ATG8 and G co-expressed with an empty vector (EV), N, P, P3, M, L or P6. Bars, 5 µm. (**B**) Average number of discrete puncta of GFP.ATG8 in Sf9 cells, as measured in 30 cells, *p< 0.05, **p< 0.01, ***p< 0.001.
(TIF)

S7 Fig RSMV M protein suppresses autophagy to promote viral replication in *R. dorsalis.* (**A-C**) Relative transcript levels for *AMPK*, *ATG14*, *BECN1*, *VPS34*(**A**), *ATG8*, *LAMP1*, *SQSTM1* (B), RSMV- *N,* –*P* and –*M* (C) in viruliferous insects injected with M or without M, as measured by RT-qPCR assay. Means (± SE) from three biological replicates are shown. (**D**) Relative intensities of bands for ATG8, AMPK, BECN1, p-BECN1,ATG14, SQSTM1, LAMP1, and RSMV-P are shown below. GAPDH was used as a control. Data are representative of three biological replicates. G, G protein injected. (**E**) Relative intensities of bands for AMPK, BECN1,ATG14, ATG8, SQSTM1 and LAMP1 are shown below. GAPDH was used as a control. Data are representative of three biological replicates. (**F**) PI3P content in nonviruliferous or viruliferous *R. dorsalis*, as determined using a PI3P ELISA assay kit. Data are representative of three biological replicates. M+, M protein injected, M-, M protein non-injected.ns, nosignificant, **P < 0.01, ***P < 0.001.
(TIF)

S8 Fig RT-qPCR analysis of mRNA transcription levels of autophagy-related genes and RSMV genes in *R. dorsalis* infected with RSMV. RT-qPCR assays showing the effect of RSMV infection on the mRNA expression levels of *ATG8* (A), *LAMP1*(B), *SQSTM1*(C), M and G (D) from 4-12-day dpi. Thirty nonviruliferous or viruliferous leafhoppers were used for RT-qPCR assays. Expression levels were normalized against the EEF1A1 transcript expression level. Transcript levels of genes in random nonviruliferous leafhoppers at 4-day padp were normalized to 1. Data are presented as mean (± SE). *P<0.05, **P<0.01, ***P<0.001.
(TIF)

S9 Fig The interactions of ATG14/BECN1, ATG14/VPS34 and BECN1/VPS34. (A, B, D, and E) Y2H assays showing that the interactions of ATG14/BECN1 (A), ATG14-N/BECN1 (B), ATG14/VPS34 (D) and BECN1/VPS34(E). Transformants on SD/-Trp-Leu-Ade-His plates are labeled as follows: +, positive control (pGBKT7–53/pGADT7-T); –, negative control (pGBKT7-Lam/pGADT7-T); DDO, SD -Trp -Leu medium; QDO, SD -Trp -Leu -His -Ade medium. (C and F) GST affinity-isolation assay showing interactions between ATG14-N and BECN1 (C) or BECN1 and VPS34 (F). ATG14 or VPS34 fused with GST served as the bait, GST served as the control, and BECN1 fused with His served as the prey. The baits or GST control were incubated with cell lysate expressing His-fused protein. Input and affinity-isolation samples were detected by immunoblotting using antibodies against GST or His.
(TIF)

S1 Table Raw data measured in Fig 2D. (PDF)

S2 Table Primers used in this study. (PDF)

S3 Table Plasmid constructs used in the study. (PDF)
