# Supplementary figures and images for: Rice stripe mosaic virus M protein antagonizes G-protein-induced antiviral autophagy in insect vectors
Source: PLoS Pathog. 2025 Apr 29;21(4):e1013070. doi: 10.1371/journal.ppat.1013070 (PMC12040238; doi:10.1371/journal.ppat.1013070)

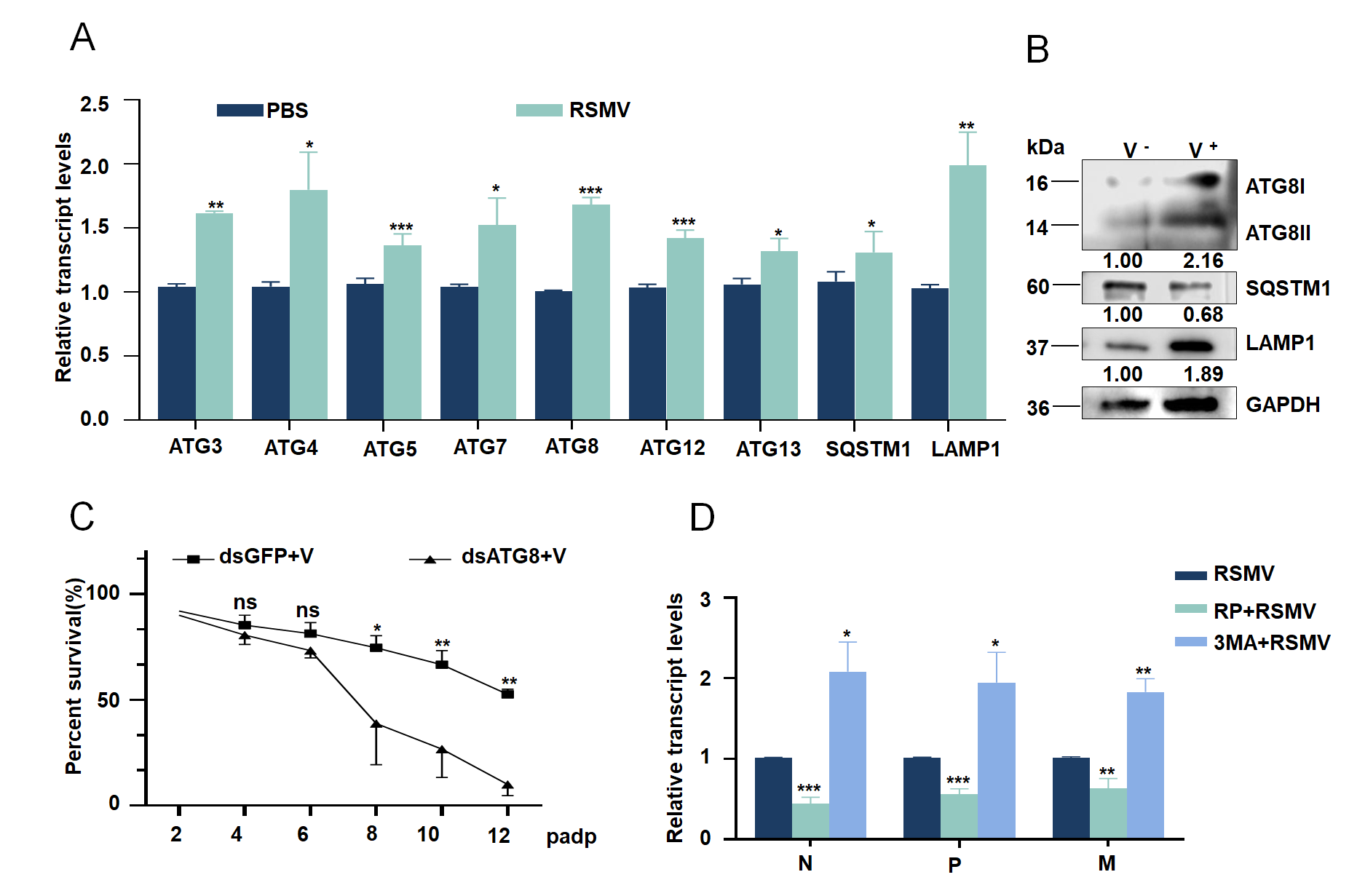

Supplement: S1 Fig — (A)Relative transcript levels for ATG3, ATG4, ATG5, ATG7, ATG8, ATG12, ATG13, SQSTM1 and LAMP1 in nonviruliferous and viruliferous insects, as measured by RT-qPCR assay. (B) The expressions of ATG8, SQSTM1 and LAMP1 in nonviruliferous or viruliferous insects as detected by western blot assay. GAPDH was detected as a control. Data are representative of three biological replicates. (C) The survival rates of viruliferous insects were evaluated after microinjection with dsGFP or dsATG8, with 100 insects per group. Means (± SD) from three biological replicates are shown. (D) The relative transcript levels of RSMV-N, -P, and -M were determined by RT-qPCR following treatments with rapamycin or 3-MA for 5 days. Means (± SE) from three biological replicates are shown. RP, rapamycin; 3-MA, 3-Methyladenine; *P < 0.05, **P < 0.01, ***P < 0.001. (TIF) [file ppat.1013070.s001.tif]

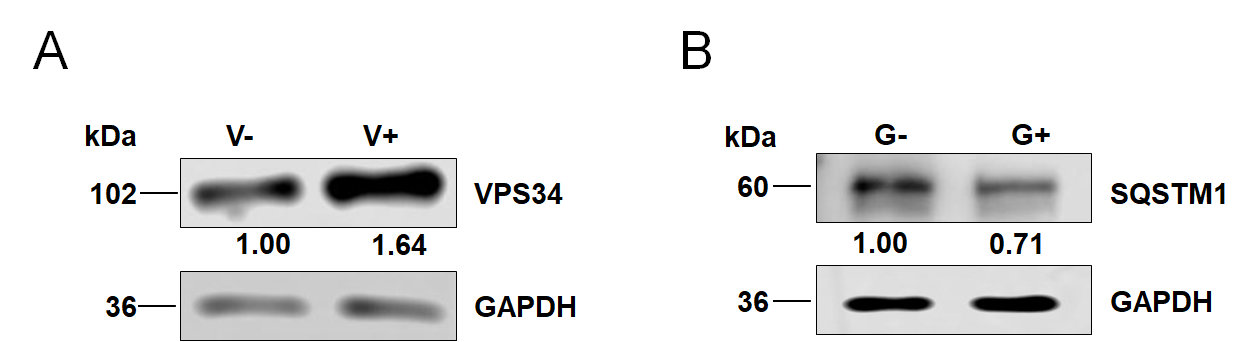

Supplement: S2 Fig — (A) RSMV infection increases the expression of VPS34 protein in R. dorsalis. Relative intensities of bands for of VPS34 are shown below. GAPDH was used as a control. Data are representative of three biological replicates. V-, nonviruliferous, V+, viruliferous. (B) The expression of SQSTM1 in R. dorsalis with the microinjection of G. GAPDH was used as a control. Data are representative of three biological replicates. G+, G protein injected, G-, G protein non-injected. (TIF) [file ppat.1013070.s002.tif]

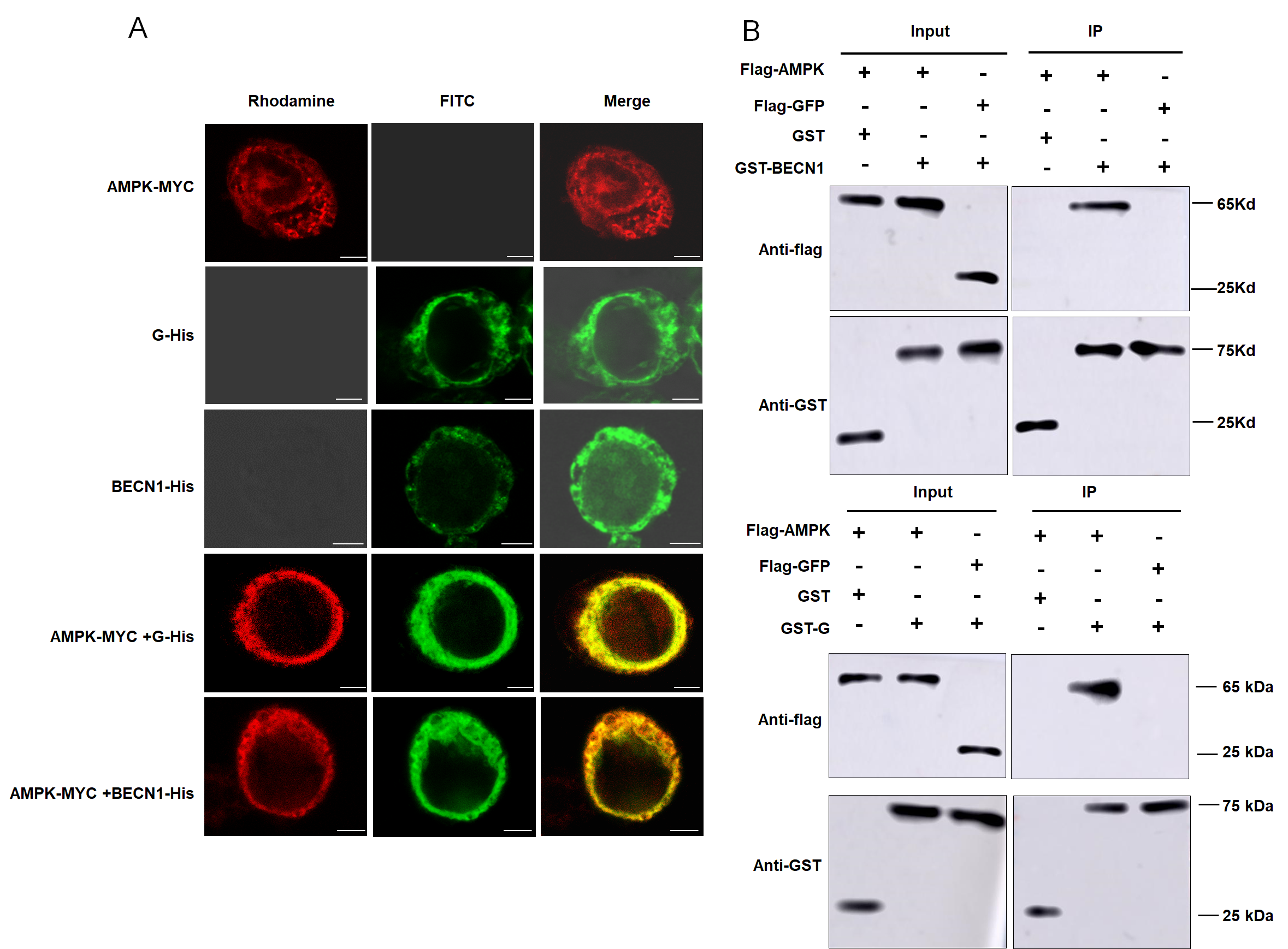

Supplement: S3 Fig — Sf9 cells were fixed at 48 hpi and immunolabeled with AMPK- rhodamine (red), BECN1-FITC (green), or G-FITC (green). The images showing single expression or co-expression were merged under abackground of transmitted light. Bars: 10 um. (B) Co-IP assays examining the interactions of AMPK/BECN1 and AMPK/G in vivo. At 3 days post-infiltration, total proteins extracted Sf9 cells expressing indicated proteins were precipitated with anti-GST beads and analyzed by western blotting with anti-GST and anti-Flag antibodies. (TIF) [file ppat.1013070.s003.tif]

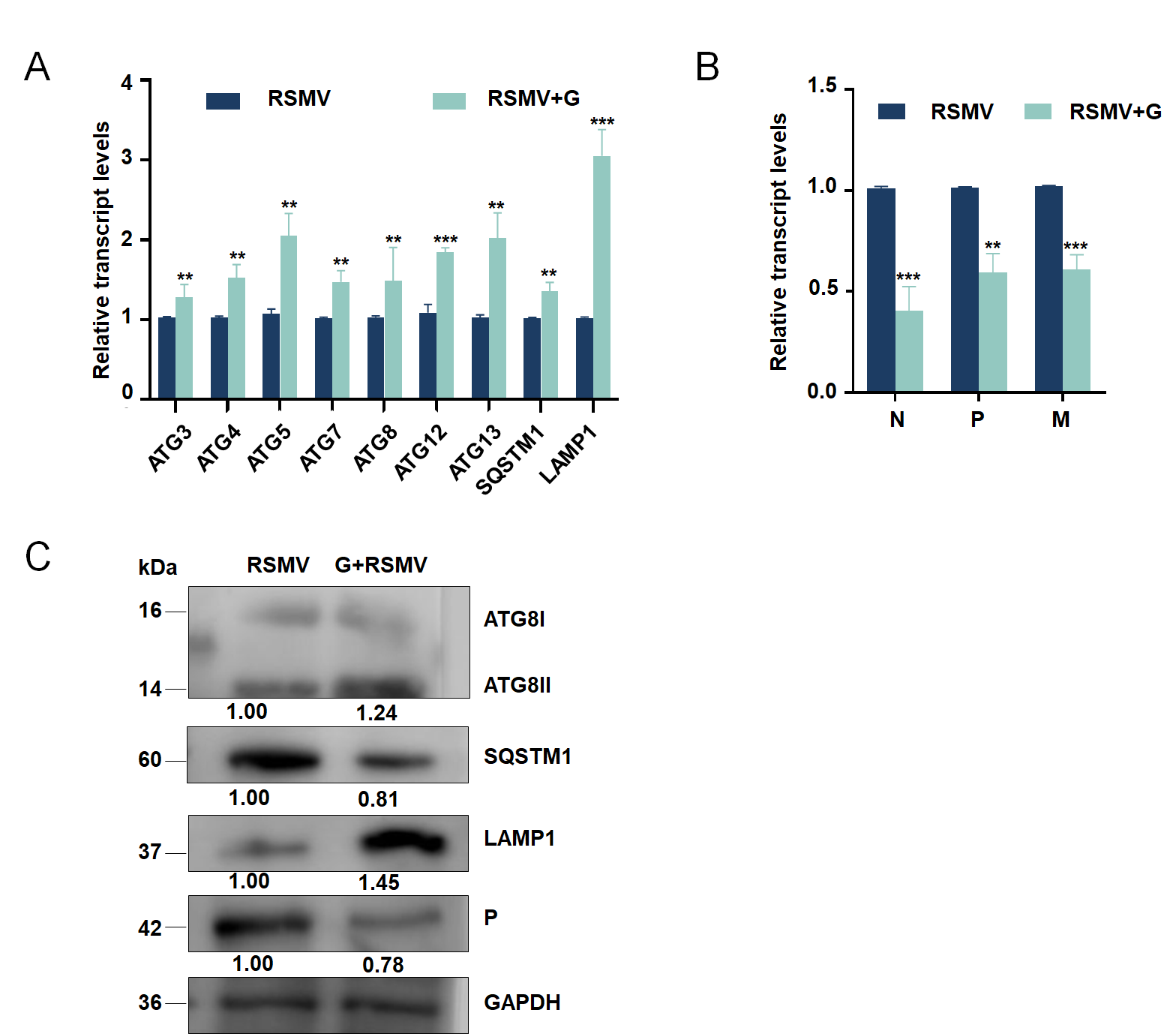

Supplement: S4 Fig — (A, B)Relative transcript levels for ATG3, ATG4, ATG5, ATG7, ATG8, ATG12, ATG13, SQSTM1, LAMP1 (A), RSMV-N, –P and -M (B) in viruliferous insects injected with G or without G, as measured by RT-qPCR assay. Means (± SE) from three biological replicates are shown. (C) Relative intensities of bands for ATG8, SQSTM1, LAMP1, and RSMV-P are shown below. GAPDH was used as a control. Data are representative of three biological replicates. G, G protein injected; **P < 0.01, ***P < 0.001. (TIF) [file ppat.1013070.s004.tif]

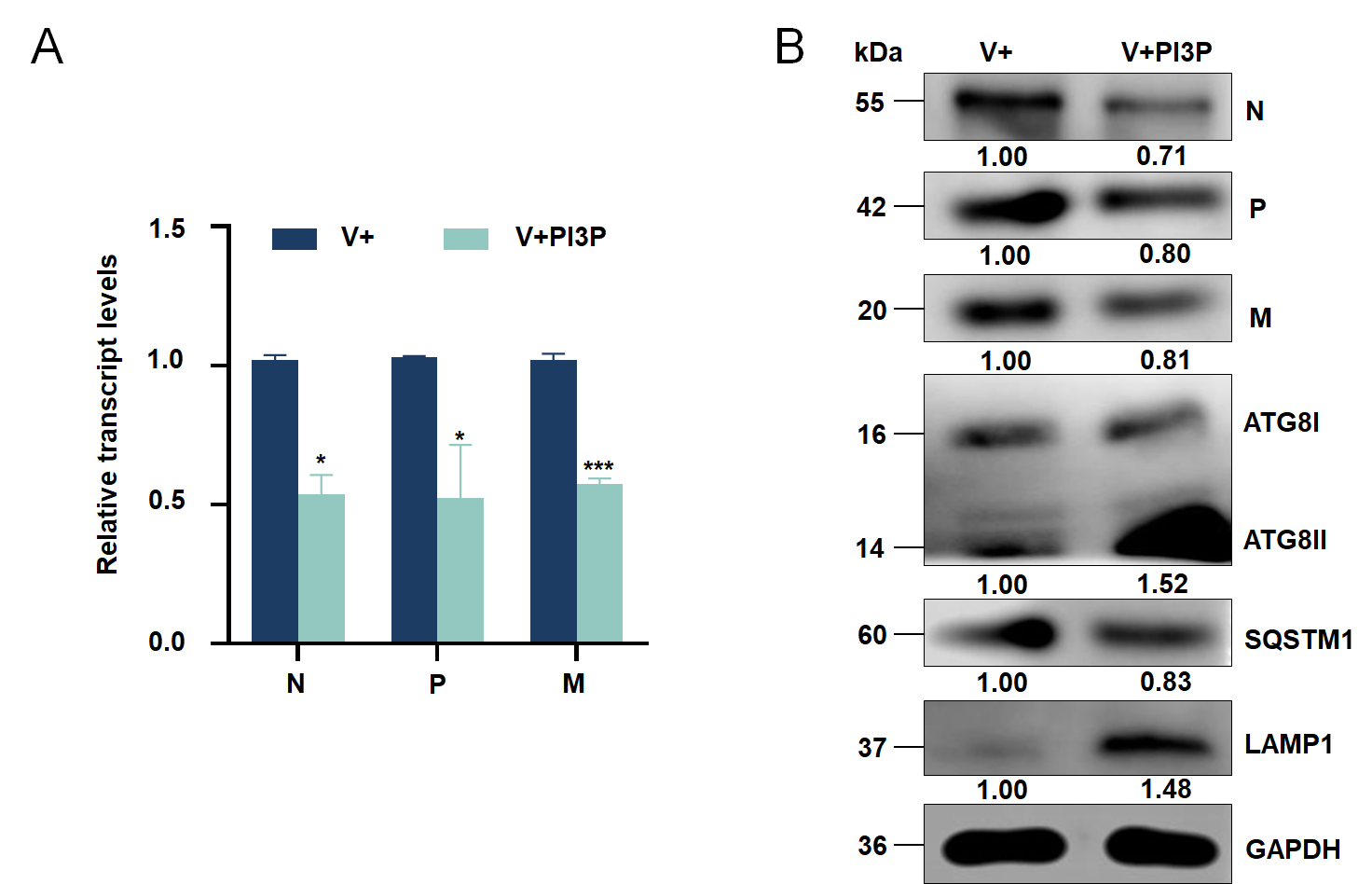

Supplement: S5 Fig — (A)The relative transcript levels of RSMV-N, -P, and -M were determined by RT-qPCR following microinjected with PI3P or without PI3P for 7 days. Means (± SE) from three biological replicates are shown. Data are presented as mean (± SE). *P<0.05, ***P<0.001. (B) Relative intensities of bands for of RSMV-N, -P, -MATG8, SQSTM1 and LAMP1 are shown below. GAPDH was used as a control. Data are representative of three biological replicates. (TIF) [file ppat.1013070.s005.tif]

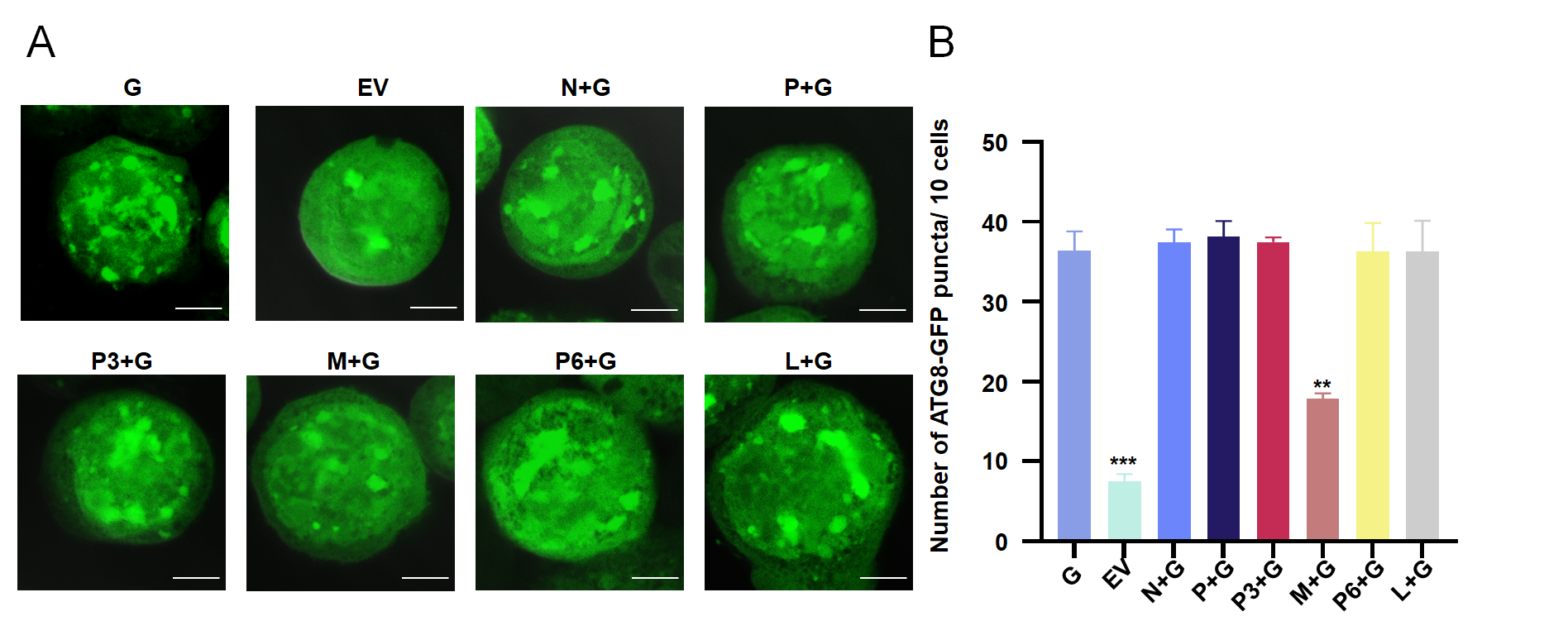

Supplement: S6 Fig — (A) Immunofluorescence assay showing GFP-ATG8 (green) in sf9 cells co-expressed with G as a control, or GFP-ATG8 and G co-expressed with an empty vector (EV), N, P, P3, M, L or P6. Bars, 5 µm. (B) Average number of discrete puncta of GFP.ATG8 in Sf9 cells, as measured in 30 cells, *p< 0.05, **p< 0.01, ***p< 0.001. (TIF) [file ppat.1013070.s006.tif]

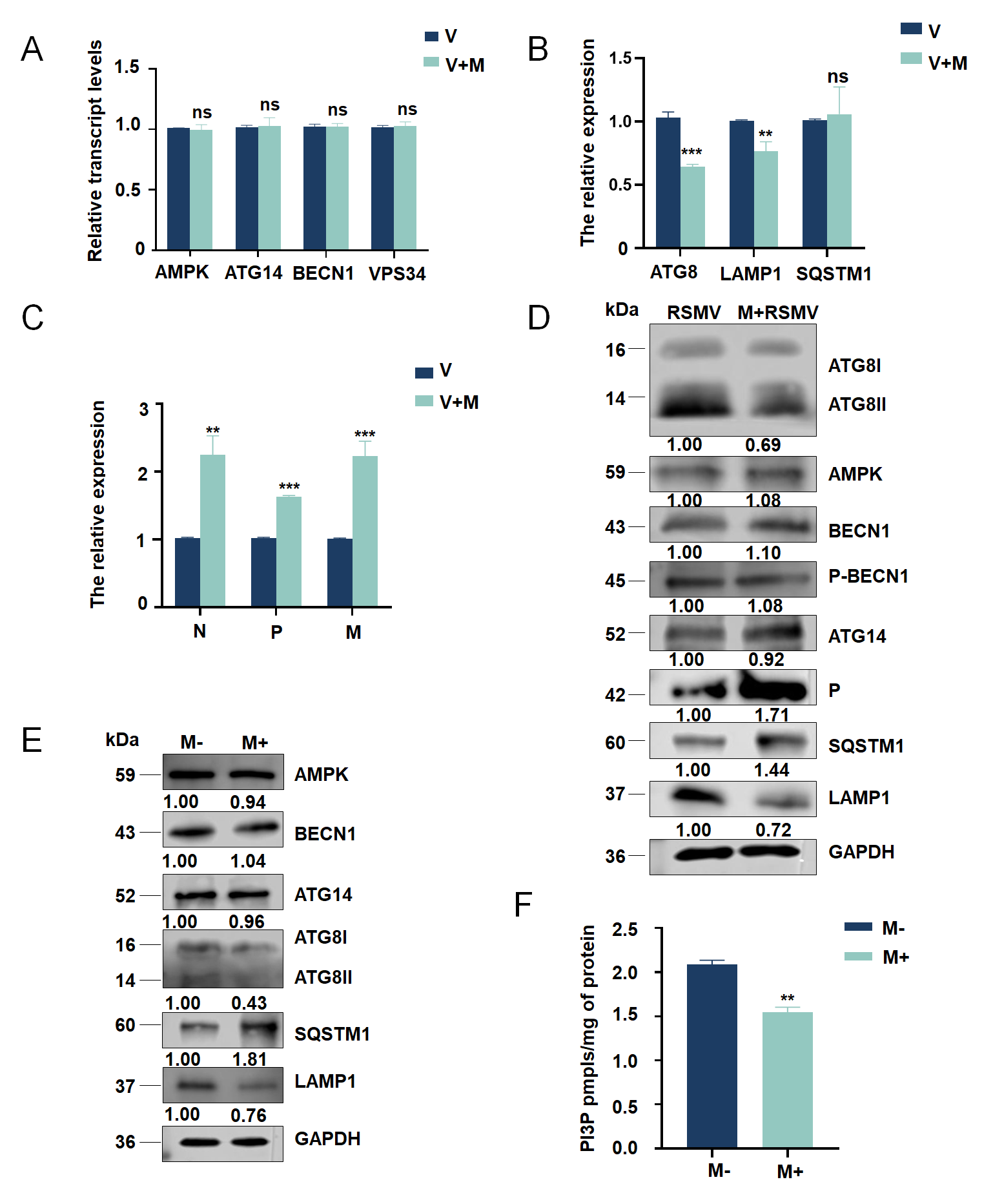

Supplement: S7 Fig — (A-C) Relative transcript levels for AMPK, ATG14, BECN1, VPS34(A), ATG8, LAMP1, SQSTM1 (B), RSMV- N, –P and –M (C) in viruliferous insects injected with M or without M, as measured by RT-qPCR assay. Means (± SE) from three biological replicates are shown. (D) Relative intensities of bands for ATG8, AMPK, BECN1, p-BECN1,ATG14, SQSTM1, LAMP1, and RSMV-P are shown below. GAPDH was used as a control. Data are representative of three biological replicates. G, G protein injected. (E) Relative intensities of bands for AMPK, BECN1,ATG14, ATG8, SQSTM1 and LAMP1 are shown below. GAPDH was used as a control. Data are representative of three biological replicates. (F) PI3P content in nonviruliferous or viruliferous R. dorsalis, as determined using a PI3P ELISA assay kit. Data are representative of three biological replicates. M+, M protein injected, M-, M protein non-injected.ns, nosignificant, **P < 0.01, ***P < 0.001. (TIF) [file ppat.1013070.s007.tif]

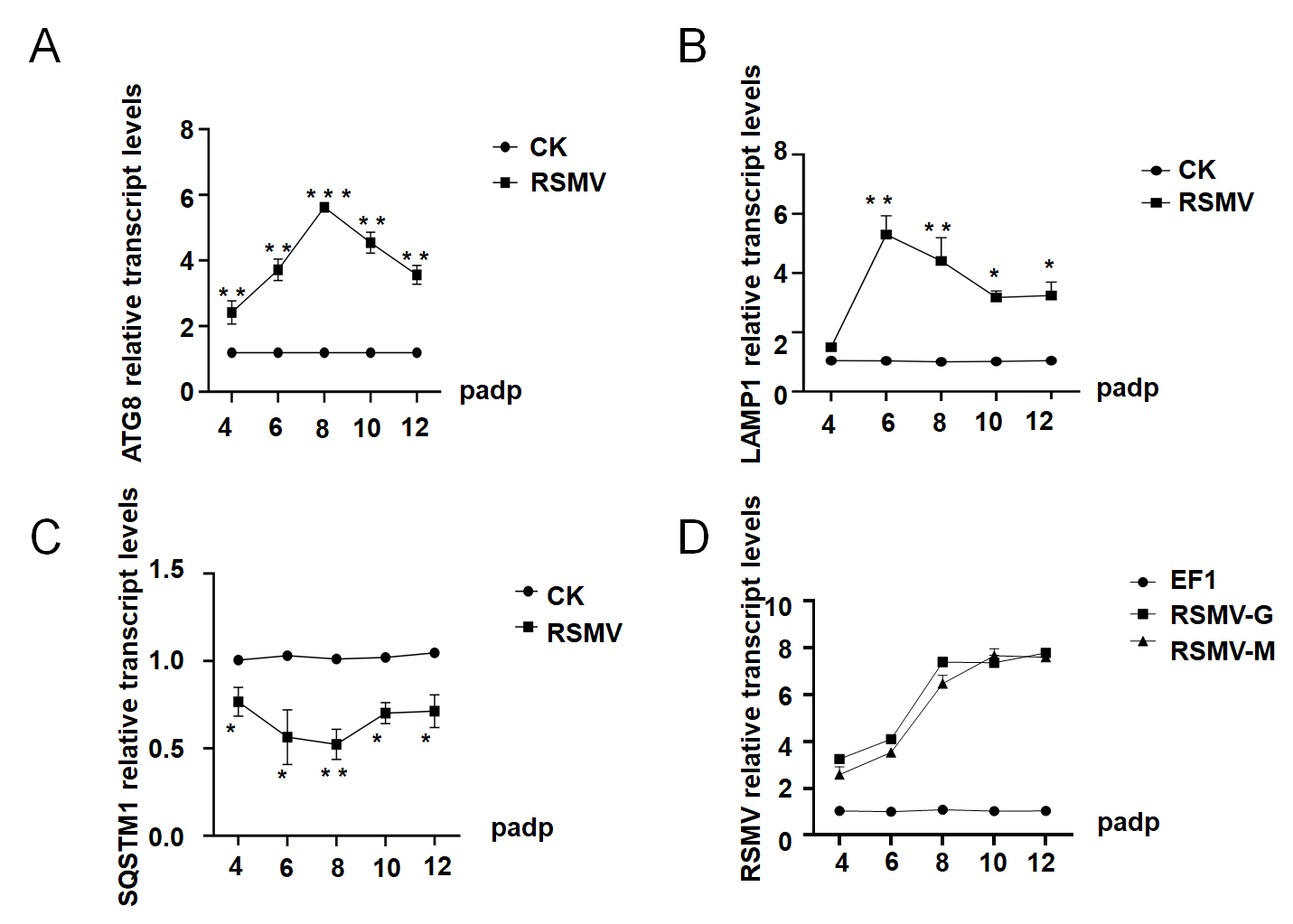

Supplement: S8 Fig — RT-qPCR assays showing the effect of RSMV infection on the mRNA expression levels of ATG8 (A), LAMP1(B), SQSTM1(C), M and G (D) from 4-12-day dpi. Thirty nonviruliferous or viruliferous leafhoppers were used for RT-qPCR assays. Expression levels were normalized against the EEF1A1 transcript expression level. Transcript levels of genes in random nonviruliferous leafhoppers at 4-day padp were normalized to 1. Data are presented as mean (± SE). *P<0.05, **P<0.01, ***P<0.001. (TIF) [file ppat.1013070.s008.tif]

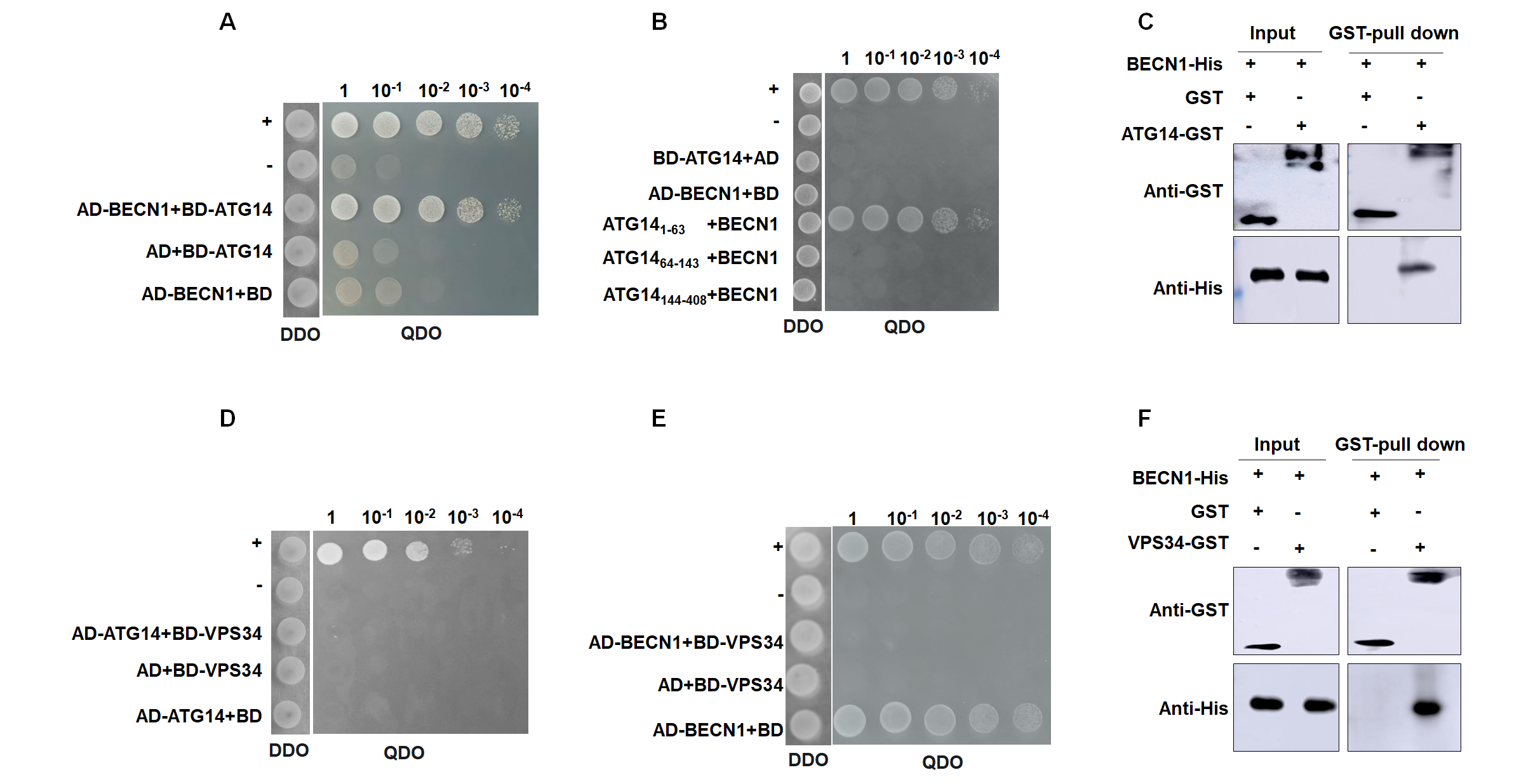

Supplement: S9 Fig — (A, B, D, and E) Y2H assays showing that the interactions of ATG14/BECN1 (A), ATG14-N/BECN1 (B), ATG14/VPS34 (D) and BECN1/VPS34(E). Transformants on SD/-Trp-Leu-Ade-His plates are labeled as follows: +, positive control (pGBKT7–53/pGADT7-T); –, negative control (pGBKT7-Lam/pGADT7-T); DDO, SD -Trp -Leu medium; QDO, SD -Trp -Leu -His -Ade medium. (C and F) GST affinity-isolation assay showing interactions between ATG14-N and BECN1 (C) or BECN1 and VPS34 (F). ATG14 or VPS34 fused with GST served as the bait, GST served as the control, and BECN1 fused with His served as the prey. The baits or GST control were incubated with cell lysate expressing His-fused protein. Input and affinity-isolation samples were detected by immunoblotting using antibodies against GST or His. (TIF) [file ppat.1013070.s009.tif]
