# Supplementary material for: Rice stripe mosaic virus M protein antagonizes G-protein-induced antiviral autophagy in insect vectors
Source: PLoS Pathog. 2025 Apr 29;21(4):e1013070. doi: 10.1371/journal.ppat.1013070 (PMC12040238; doi:10.1371/journal.ppat.1013070)
Supplement: S1 Table — (PDF) [file ppat.1013070.s010.pdf]

S1 Table. Raw data measured in Fig 2D

OD	PI3P pmpls/mg
2.141328211	2.60949506
2.09467618	2.436608123
2.026070252	2.568331504
1.891602634	2.14571899
1.543084523	2.263721186
2.053512623	2.299396268
