# Supplementary material for: Rice stripe mosaic virus M protein antagonizes G-protein-induced antiviral autophagy in insect vectors
Source: PLoS Pathog. 2025 Apr 29;21(4):e1013070. doi: 10.1371/journal.ppat.1013070 (PMC12040238; doi:10.1371/journal.ppat.1013070)
Supplement: S2 Table — (PDF) [file ppat.1013070.s011.pdf]

S2 Table. Primers used in this study.	
**Primer name**	**5'-3'**
qRSMV-N-F	TGTCACAGAGTCATGGGAGC
qRSMV-N-R	CAGCCTCGGCTGTAGATTGA
qRSMV-P-F	TGACTGAGCTGCAGGGGATA
qRSMV-P-R	CGGCTTGGAGATGTCCACTT
qRSMV-M-F	CCGAGTGACAGGAGGATCAC
qRSMV-M-R	CATTCTCCTGGTAGGCGGAC
qEF1-F	AGGGCATCTTGTCAGAGGGC
qEF1-R	GAGAAAAGGAAAGCCGAGGGC
qATG8-F	GAGAAAAGGAAAGCCGAGGGC
qATG8-R	GGTCTGAGGTGAACTCGCT
qATG14-F	TCCACTTTGTTGCAAATCCAGG
qATG14-R	AGAAGTCGCCACTCAAGCAA
qVPS34-F	CCGTATGGAATCGCACCTGA
qVPS34-R	ACTCAACTTCATGGGCGGAG
qBECN1-F	AGCCGACACAGACGAGACTA
qBECN1-R	ATGGTCCCAAAGTGACCGTG
qAMPK-F	TAGGACAAACTCTGGGCGTG
qAMPK-R	ACGCCTAATCTTCCCCACAA
AMPK-F	ATGGGGGAGAAGTCACAGGG
AMPK-R	TCAGCGTGCCAGCTGAGTAA
VPS34-F	ATAGTAGGATTAACGATTGCT
VPS34-R	TTAGTTCCGCCAATACTGT
BECN1-F	ATGGTTCCGGCGTTCAG
BECN1-R	CTACTCAATCTGATCTTTGGC
ATG14-F	ATGGCATCAATTACACCTAT
ATG14-R	TTATCTGTTGGTGGACCAG
Pet28b-M-F	AGCAAATGGGTCGGGATCCGATGGCCGTTCCGTGGACT
Pet28b-M-R	TCGAGTGCGGCCGCAAGCTTTTATACTTCCTCCATCCACCTTGC
Pet28b-ATG14-F	AGCAAATGGGTCGGGATCCGATGGCATCAATTACACCTAT
Pet28b-ATG14-R	TCGAGTGCGGCCGCAAGCTTTCTGTTGGTGGACCAG
Pet28b-BECN1-F	AGCAAATGGGTCGGGATCCGATGGTTCCGGCGTTCAG
Pet28b-BECN1-R	TCGAGTGCGGCCGCAAGCTTCTCAATCTGATCTTTGGC

pGEX4T-3-ATG14-F	CGGATCTGGTTCCGCGTGGAATGGCATCAATTACACCTATTCAT
pGEX4T-3-ATG14-R	ACGATGCGGCCGCTCGAGTCTCTGTTGGTGGACCAG
PGEX4T-3-AMPK-F	CGCGTGGATCCCCGAATTCCATGGGGGAGAAGTCACAGGG
PGEX4T-3-AMPK-R	ACGATGCGGCCGCTCGAGTCTCAGCGTGCCAGCTGAGTAA
PGEX4T-3-VPS34-F	CGCGTGGATCCCCGAATTCCATGGTAGGATTAACGATTGCT
PGEX4T-3-VPS34-R	ACGATGCGGCCGCTCGAGTCGTTCCGCCAATACTGT
pFAST-BECN1-his-F	GTCCGAAGCGCGCGGATGGTTCCGGCGTTCAG
pFAST-BECN1-his-R	GTACTTCTCGACAttaATGGTGATGGTGATGATGCTCAATCTGATCTTTGGC
pFAST-ATG14-strep-F	CCCGGTCCGAAGCGCGCGGAATTCAATGGCATCAATTACACCTATTCAT
pFAST-ATG14-strep-R	CTAGTACTTCTCGACAAGCTTTTATTTTTCGAACTGCGGGTGGCTCCATCTGTTGGTGGACCAG
dsAMPK-F	ATTCTCTAGAAGCTTAATACGACTCACTATAGGGATTATGGAGTATGTGTCTGG
dsAMPK-R	ATTCTCTAGAAGCTTAATACGACTCACTATAGGGAGGATGGGGTTTTATTGGG
dsATG14-F	ATTCTCTAGAAGCTTAATACGACTCACTATAGGGCTGCACTGGCCCGATCCT
dsATG14-R	ATTCTCTAGAAGCTTAATACGACTCACTATAGGGCTGCACTGGCCCGATCCT
dsATG8-F	ATTCTCTAGAAGCTTAATACGACTCACTATAGGGGTGATCTGGACAAGAAGA
dsATG8-R	ATTCTCTAGAAGCTTAATACGACTCACTATAGGGAGCATCCCCGTAGACATT
dsBECN1-F	ATTCTCTAGAAGCTTAATACGACTCACTATAGGGTGGGTCGACTGCCA
dsBECN1-R	ATTCTCTAGAAGCTTAATACGACTCACTATAGGGCTCAATCTGATCTTTGGC
AD-VPS34-F	CATGGAGGCCAGTGAAATAGTAGGATTAACGATTGCT
AD-VPS34-R	AGCTCGATGGATCCCTTAGTTCCGCCAATACTGT
AD-ATG14-F	CATGGAGGCCAGTGAAATGGCATCAATTACACCTAT
AD-ATG14-R	AGCTCGATGGATCCCTTATCTGTTGGTGGACCAG
BD-AMPK-F	CATATGGCCATGGAGGCCGAATTCATGGGGGAGAAGTCACAGGG

BD-AMPK-R	CGGCCGCTGCAGGTCGACGGATCCTCAGCGTGCCAGCTGAGTAA	23
BD-BECN1-F	ATCTCAGAGGAGGACCTGCATATGGTTCCGGCGTTCAG	
BD-BECN1-R	CTGCAGGTCGACGGATCCTACTCAATCTGATCTTTGGC	
BD-ATG14$_{46-93}$-F	CATATGGCCATGGAGGCCGAATTCATGAATTTATCTTCTTCATC	
BD-ATG14$_{46-93}$-R	CGGCCGCTGCAGGTCGACGGATCCAGATGATGAGGATAT	
BD-ATG14$_{190-429}$-F	CATATGGCCATGGAGGCCGAATTCATGGCAGAAAGATATGCAGA	
BD-ATG14$_{190-429}$-R	CGGCCGCTGCAGGTCGACGGATCCGCTTTTCTCATTGTCAC	
BD-ATG14$_{1183-1224}$-F	CATATGGCCATGGAGGCCGAATTCATGGACACTTCGAGTGACG	
BD-ATG14$_{1183-1224}$-R	CGGCCGCTGCAGGTCGACGGATCCGTCACTGTCTTCTTCCG	
