# Supplementary material for: Rice stripe mosaic virus M protein antagonizes G-protein-induced antiviral autophagy in insect vectors
Source: PLoS Pathog. 2025 Apr 29;21(4):e1013070. doi: 10.1371/journal.ppat.1013070 (PMC12040238; doi:10.1371/journal.ppat.1013070)
Supplement: S3 Table — (PDF) [file ppat.1013070.s012.pdf]

**S3 Table. Plasmid constructs used in the study.**		
Plasmid name	Vector	Purpose
BD-AMPK	pGBKT7	Expression in *S. cerevisiae* for yeast two hybird
BD-AMPK	pGBKT7	Expression in *S. cerevisiae* for yeast two hybird
BD-BECN1	pGBKT7	Expression in *S. cerevisiae* for yeast two hybird
BD-BECN1	pGBKT7	Expression in *S. cerevisiae* for yeast two hybird
BD-ATG14$_{46\text{-}93}$	pGBKT7	Expression in *S. cerevisiae* for yeast two hybird
BD-ATG14$_{46\text{-}93}$	pGBKT7	Expression in *S. cerevisiae* for yeast two hybird
BD-ATG14$_{190\text{-}429}$	pGBKT7	Expression in *S. cerevisiae* for yeast two hybird
BD-ATG14$_{190\text{-}429}$	pGBKT7	Expression in *S. cerevisiae* for yeast two hybird
BD-ATG14$_{1183\text{-}1224}$	pGBKT7	Expression in *S. cerevisiae* for yeast two hybird
BD-ATG14$_{1183\text{-}1224}$	pGBKT7	Expression in *S. cerevisiae* for yeast two hybird
AD-VPS34	pGADT7	Expression in *S. cerevisiae* for yeast two hybird
AD-VPS34	pGADT7	Expression in *S. cerevisiae* for yeast two hybird
AD-ATG14	pGADT7	Expression in *S. cerevisiae* for yeast two hybird
AD-ATG14	pGADT7	Expression in *S. cerevisiae* for yeast two hybird
Pet28b-M	Pet28b	Expression in *E. coli* for *in vitro* pulldown
Pet28b-M	Pet28b	Expression in *E. coli* for *in vitro* pulldown
Pet28b-ATG14	Pet28b	Expression in *E. coli* for *in vitro* pulldown
Pet28b-ATG14	Pet28b	Expression in *E. coli* for *in vitro* pulldown
Pet28b-BECN1	Pet28b	Expression in *E. coli* for *in vitro* pulldown
Pet28b-BECN1	Pet28b	Expression in *E. coli* for *in vitro* pulldown
pGEX4T-3-ATG14	pGEX4T-3	Expression in *E. coli* for *in vitro* pulldown
pGEX4T-3-ATG14	pGEX4T-3	Expression in *E. coli* for *in vitro* pulldown
PGEX4T-3-AMPK	pGEX4T-3	Expression in *E. coli* for *in vitro* pulldown
PGEX4T-3-AMPK	pGEX4T-3	Expression in *E. coli* for *in vitro* pulldown
PGEX4T-3-VPS34	pGEX4T-3	Expression in *E. coli* for *in vitro* pulldown
PGEX4T-3-VPS34	pGEX4T-3	Expression in *E. coli* for *in vitro* pulldown
pFAST-BECN1-his	pFAST-Bac1	Transient expression in Sf9 cells
pFAST-BECN1-his	pFAST-Bac1	Transient expression in Sf9 cells
pFAST-ATG14-strep	pFAST-Bac1	Transient expression in Sf9 cells
pFAST-ATG14-strep	pFAST-Bac1	Transient expression in Sf9 cells
